# NFAT1 and NFκB regulates expression of the common γ-chain cytokine receptor in activated T cells

**DOI:** 10.1186/s12964-023-01326-7

**Published:** 2023-10-30

**Authors:** Ju A Shim, So Min Lee, Jin Woo Jeong, Hyori Kim, Woo Jae Son, Jun Hong Park, Parkyong Song, Sin-Hyeog Im, Sangsu Bae, Jung-Hyun Park, Yuna Jo, Changwan Hong

**Affiliations:** 1 Department of Anatomy, Pusan National University School of Medicine, Room 504, 49 Busandaehak-Ro, Yangsan, Gyeongsangnam-Do 50612 South Korea; 2 Department of Convergence Medical Science, Pusan National University School of Medicine, Yangsan, 50612 Republic of Korea; 3 PNU GRAND Convergence Medical Science Education Research Center, Pusan National University School of Medicine, Yangsan, 50612 Republic of Korea; 4 Department of Chemistry, Hanyang University, Seoul, 04763 Republic of Korea; 5 Herbal Medicine Resources Research Center, Korea Institute of Oriental Medicine, Naju, 58245 Republic of Korea; 6 University of Science & Technology (UST), KIOM Campus, Korean Convergence Medicine Major, Daejeon, 34054 Republic of Korea; 7 Department of Life Sciences, Pohang University of Science and Technology (POSTECH), Pohang, 37673 Korea; 8 Department of Biomedical Sciences, Seoul National University College of Medicine, Seoul, 03080 Republic of Korea; 9 Experimental Immunology Branch, Center for Cancer Research, National Cancer Institute, NIH, Bethesda, MD 20892 USA; 10 Department of Anatomy, Pusan National University School of Medicine, Room 515, 49 Busandaehak-Ro, Yangsan, Gyeongsangnam-Do 50612 South Korea

**Keywords:** Common gamma chain, TCR signaling, NFAT1, NFκB, T cell activation

## Abstract

**Introduction:**

Cytokines of the common γ chain (γc) family are critical for the development, differentiation, and survival of T lineage cells. Cytokines play key roles in immunodeficiencies, autoimmune diseases, allergies, and cancer. Although γc is considered an assistant receptor to transmit cytokine signals and is an indispensable receptor in the immune system, its regulatory mechanism is not yet well understood.

**Objective:**

This study focused on the molecular mechanisms that γc expression in T cells is regulated under T cell receptor (TCR) stimulation.

**Methods:**

The γc expression in TCR-stimulated T cells was determined by flow cytometry, western blot and quantitative RT-PCR. The regulatory mechanism of γc expression in activated T cells was examined by promoter-luciferase assay and chromatin immunoprecipitation assays. NFAT1 and NFκB deficient cells generated using CRISPR-Cas9 and specific inhibitors were used to examine their role in regulation of γc expression. Specific binding motif was confirmed by γc promotor mutant cells generated using CRISPR-Cas9. IL-7TgγcTg mice were used to examine regulatory role of γc in cytokine signaling.

**Results:**

We found that activated T cells significantly upregulated γc expression, wherein NFAT1 and NFκB were key in transcriptional upregulation via T cell receptor stimulation. Also, we identified the functional binding site of the γc promoter and the synergistic effect of NFAT1 and NFκB in the regulation of γc expression. Increased γc expression inhibited IL-7 signaling and rescued lymphoproliferative disorder in an IL-7Tg animal model, providing novel insights into T cell homeostasis.

**Conclusion:**

Our results indicate functional cooperation between NFAT1 and NFκB in upregulating γc expression in activated T cells. As γc expression also regulates γc cytokine responsiveness, our study suggests that γc expression should be considered as one of the regulators in γc cytokine signaling and the development of T cell immunotherapies.

Video Abstract

**Supplementary Information:**

The online version contains supplementary material available at 10.1186/s12964-023-01326-7.

## Introduction

The common γ-chain (γc) family of cytokines, which comprises interleukin (IL)-2, IL-4, IL-7, IL-9, IL-15, and IL-21, depends on the shared γc receptor subunit for cytokine signaling and is involved in the development, differentiation, and homeostasis of various immune cell types [1, 2]. γc deficiency leads to X-linked severe combined immunodeficiency (X-SCID) in both humans and mice [3]. γc family cytokine responsiveness is regulated by altered cytokine-specific receptor expression, including that of IL-2Rα/β, IL-4Rα, IL-7Rα, IL-9Rα, IL-15Rα, and IL-21Rα [1]. It is generally speculated that γc is constantly expressed and is an accessory protein that transmits cytokine signals [1]. Thus, the signals of the γc family of cytokines may not be controlled by γc. For this reason, to the best of our knowledge, studies on γc have not been conducted, and the properties of γc expression, regulatory mechanisms, and related factors remain little understood. However, γc expression is negatively regulated during the double-positive stage of thymic development [4], and a new γc mRNA splice isoform has been identified in both mouse and human T cells [5]. The γc splice isoform (sγc) encodes a truncated γc protein that is soluble, potentially secreted, and functions as a regulator of γc cytokine signaling [5]. The expression of sγc is upregulated in activated T cells, and upregulated sγc expression blocks IL-2 and IL-15 signaling in CD8^+^ T cells, attenuating CD8^+^ T cell responses to tumors [6]. This indicates that γc expression may be actively regulated and that its regulation may contribute to T cell immune responses.

The Ca^2+^-dependent nuclear factor of activated T cells (NFAT) and the PKCθ-dependent nuclear factor kappa-light-chain-enhancer of activated B cells (NFκB) are transcription factors (TFs) that mediate immune response in T lymphocytes [7–9]. NFκB forms mostly heterodimers with p65, RelB, and cRel, which have a transactivation domain, and p50 (p105; NFκB1) and p52 (p100; NFκB2) in the cytoplasm of resting T cells [10]. Upon T cell receptor (TCR) engagement, the kinase IΚΚβ phosphorylates IΚΚα, which inhibits NFκB translocation upon binding, and the phosphorylated IΚΚα is degraded by the proteasome [7]. Consequently, free NFκB is translocated into the nucleus where it initiates the transcription of genes required for the proliferation, differentiation, and pro-inflammatory functions of T cells [11, 12]. The NFAT TFs family comprises five subfamilies: NFAT1 (NFATc2), NFAT2 (NFATc1), NFAT3 (NFATc4), NFAT4 (NFATc3), and NFAT5 [9]. NFAT1, 2, and 4, whose functions are dependent on calcium/calcineurin, play critical roles [10] in T cell development, differentiation, and function [9, 13]. They have two distinct domains: the NFAT-homology region, which contains calcineurin-docking sites and several phosphorylation sites, and the Rel homology region, which contains a DNA-binding domain and AP1 contact sites [9, 14, 15]. In activated T cells, calcium released from intracellular stores binds to calmodulin, thereby activating the calmodulin-dependent phosphatase calcineurin [9]. NFATs are dephosphorylated by activated calcineurin, resulting in their translocation to the nucleus because of full exposure to a nuclear localization signal [9, 14]. In the nucleus, NFAT regulates the expression of genes involved in T cell development, activation, and differentiation [8, 9].

Although it is known that γc expression is regulated in thymocyte developmental stages [16–18] and that sγc generation is enhanced in activated T cells [4], the regulatory mechanisms and related regulatory factors have not been fully determined. In this study, we found that γc expression was transcriptionally upregulated in activated T cells and identified the functional promoter regions and TFs responsible for the regulation of γc expression in T cells. NFAT1 and NFκB, which are mainly related to TCR signaling, contributed to the upregulation of γc expression upon TCR stimulation. As γc cytokines are closely involved in autoimmune diseases and cancers, this study on the regulatory mechanisms of γc provides therapeutic benefits for these often-fatal diseases.

## Materials and methods

### Animals

C57BL/6 (B6) mice and IL-2Rγ^null^ (γcKO) were obtained from The Jackson Laboratory (Bar Harbor, ME, USA), and BALB/c mice from Charles River Laboratories (Wilmington, MA, USA). The generation of γc transgenic (Tg) mice has been previously described [5]. γcKO mice were bred with γcTg mice to generate γcKOγcTg mice. Transgenic mice expressing IL-7 (IL-7Tg) under the control of mouse H2-Eα promoter were obtained from The Jackson Laboratory [19]. We then crossed γcTg mice with the IL-7 overexpressing animal model to generate IL-7TgγcTg mice. All mice were maintained and bred in a specific pathogen-free animal facility at the Pusan National University School of Medicine. All experimental protocols were approved by the Pusan National University Institutional Animal Care and Use Committee (PNU-2020–2714).

### Isolation of T cells from lymph nodes (LNs)

LNs were collected from mice and minced to isolate LN cells. Total LN cells were incubated with BioMag goat α-mouse IgG beads (Qiagen, Hilden, Germany) for 40 min on ice to isolate LN T cells via negative selection [20]. To isolate LN CD8^+^ T cells, total LN cells were incubated with α-CD4 (GK1.5) for 30 min on ice and negatively selected using the BioMag goat α-mouse IgG (Qiagen) and BioMag goat α-rabbit IgG beads (Qiagen) for 40 min on ice. The purity of the isolated total LN T and LN CD8^+^ T cells was confirmed using FACSCanto II (Becton Dickinson, Franklin Lakes, NJ, USA) and Attune NxT Flow cytometry (Thermo Fisher Scientific, Waltham, MA, USA).

### Cell culture, stimulation, and inhibitor treatment

LN T, LN CD8^+^ T cells, and EL4 cells were cultured in RPMI 1640 medium (Welgene, Gyeongsan-si, Republic of Korea), whereas HEK293 T cells were cultured in Dulbecco’s Modified Eagle Medium (Welgene) containing 10% fetal bovine serum (Gemini, West Sacramento, CA, USA), L-glutamine, 100,000 U/ml penicillin plus 100 mg/ml streptomycin (Gibco, Waltham, MA, USA), non-essential amino acids (Sigma-Aldrich, Burlington, MA, USA), sodium pyruvate (Sigma-Aldrich), and β-mercaptoethanol (Gibco). LN T cells and LN CD8^+^ T cells were stimulated with plates bound with α-TCRβ (H57-597, 1 µg/ml)/α-CD28 (37.51, 1 µg/ml) antibodies (eBioscience, San Diego, CA. USA). LN CD8^+^ T cells were stimulated with recombinant human IL-2 (10 ng/ml), IL-7 (10 ng/ml), IL-15 (100 ng/ml), or interferon (IFN)-γ (10 ng/ml) (PeproTech Inc, Cranbury, NJ, USA) or with plates bound with α-TCR in the presence of α-IL-2 antibody 10 µg/ml (PeproTech Inc.) to block IL-2 signaling. EL4 cells were stimulated with phorbol 12-myristate 13-acetate (12.5 ng/ml) (PMA; Merck Millipore, Burlington, MA, USA) and ionomycin (1 µM) (Iono; Santa Cruz Biotechnology, Dallas, Texas, USA). To identify the mechanisms involved in regulating γc expression, LN CD8^+^ T cells stimulated with plates bound with α-TCR/α-CD28 in the presence of PD98059 (10 µM) or wortmannin (1 µM), NFAT1 inhibitor (INCA6, 5 µM), and/or NFκB inhibitor (Bay11-7802, 3 µM) (all from Sigma-Aldrich). EL4 cells were pretreated with 5,6-dichloro-1-beta-D-ribofuranosylbenzimidazole (DRB, 50 µM), cycloheximide (CHX, 2 µg/ml), MG132 (500 nM), cyclosporine A (CsA, 1 µg/ml), INCA6, and/or Bay11-7802 (Bay11) for 2 hr and then stimulated with PMA/Iono for 16 hr.

### In vivo T cell stimulation

To induce acute polyclonal T cell stimulation in vivo, BALB/c mice were injected intraperitoneally with 10 μg anti-CD3 antibodies (α-CD3, 17A2) or the appropriate isotype control IgG. Anti-CD3 and isotype control antibodies were purchased from BioXCell (Lebanon, NH, USA). After 16–18 hr, LNs were harvested from overnight α-CD3 antibody- or isotype control IgG-injected mice, and T cell surface marker expression was assessed.

### Generation of CRISPR/Cas9-based knockout (KO) cell line

Guide RNA (gRNA) vectors targeting NFAT1, p65, and cRel were produced, starting with an empty expression vector. The vector was cleaved using BsaI New England (Ipswich, MA, USA) and ligated to each targeted annealed oligonucleotide. The ligation products were transformed into DH5α chemically competent cells (CP011, Enzynomics, Daejeon, Republic of Korea), and transformed DH5α *Escherichia coli* (New England Biolabs) cells were incubated at 37 °C on an LB plate containing ampicillin. An ExprepTM Plasmid SV kit (101–102, GeneAll, Seoul, Republic of Korea) was used to extract the gRNA plasmids for transfection. For transfection, 2 × 10^6^ EL4 murine lymphoma cells provided by Dr. Se-Ho Park (Korea University, South Korea) were cultured in RPMI 1640 medium supplemented with 5% antibiotics and 10% fetal bovine serum in 24-well plates. One day after seeding, Cas9 expression plasmids (750 ng) and gRNA plasmids (250 ng) were transfected using Lipofectamine™ 2000 (Invitrogen, Waltham, MA, USA). After 3 days, the genomic DNA was purified using Proteinase K extraction buffer (40 mM Tris–HCl [pH 8.0], 1% Tween-20, 0.2 mM EDTA, 10 mg of proteinase K, and 0.2% nonidet NP-40). The insertion and deletion (indel) ratios and patterns of each genomic DNA sample were analyzed using next-generation sequencing (NGS) on an Illumina Mini-Seq platform (Illumina, San Diego, CA, USA) and Cas-Analyzer (www.rgenome.net) [21]. To obtain NFAT1 KO, p65 KO, and cRel KO, we made one gene KO cells each and sequentially disrupted p65 and cRel, and NFAT1 to generate NFκB (p65 and cRel) KO and NFAT1/NFκB KO. Each targeted cell group with a high mutation ratio was spread such that one cell entered a 96-well plate. After 2 weeks, single cloned cells were analyzed using NGS, and indel patterns were identified using Cas-Analyzer.

### Flow cytometry analysis

Organs were processed into single-cell suspensions, stained, and analyzed using FACSAria or FACSCanto II (Becton Dickinson) and Attune NxT Flow cytometry (Thermo Fisher Scientific). Dead cells were excluded by forward light-scatter gating and propidium iodide staining. Data were analyzed using FlowJo version 10.3 (TreeStar). Antibodies with the indicated specificities were used for staining: CD69 (H1.2F3), γc (4G3), and human CD3 (Leu4) (BD Biosciences, Franklin Lakes, NJ, USA); IL-7Rα (A7R34), IL-2Rα (CD25), Foxp3 (FJK-16s), and CD4 (GK1.5) (eBioscience); and TCRβ (H57-597), CD8α (53-6-7), and phosphorylated STAT5 (Tyr694) (all from BioLegend, San Diego, CA, USA). An anti-mouse CD16/32 antibody (2.4G2; BioLegend) was used to block the Fc receptor. pSTAT5 expression was determined in cytokine-stimulated cells (30 min) by methanol/acetone fixation and intracellular staining [6, 22, 23]. Intranuclear Foxp3 were detected using Foxp3 staining kit according to manufacturer’s instructions (eBioscience).

### Reverse transcription-quantitative PCR (RT-qPCR)

Total RNA was extracted from cultured cells using Ribospin (GeneAll). RNA was reverse-transcribed to cDNA via oligo(dT) priming using a QuantiTect Reverse transcription kit (GeneAll). RT-qPCR was conducted using a LightCycler 96 Real-Time PCR System (Roche, Basel, Switzerland) and a SYBR Green detection system (Bio-Rad, Hercules, CA, USA). The following primer sequences were used: mγc (forward, 5'-CATGAACCTAGATTCTCCCTGCC-3'; reverse, 5'-CCAACCAACAGTACACAAAGATCAG-3'), sγc (forward, 5′-CATGAACCTAGATTCTCCCTGCC-3′; reverse, 5′- TGATGGGGGGAATTGGAGIIIIICCTCTACA-3′), IL-7Rα (forward, 5'-CACACAAGAACAACAATCCCACA-3'; reverse, 5'- GATCCCATCCTCCTTGATTCTTG-3'), and RPL13 (forward, 5'-CGAGGCATGCTGCCCCACAA-3'; reverse, 5'-AGCAGGGACCACCATCCGCT-3'). The thermal cycling conditions were as follows: 40 cycles of denaturation at 95 °C for 10 s, annealing at 59 °C for 30 s, and extension at 72 °C for 30 s. Gene expression values were normalized to those of *Rpl13* in the same sample.

### Enzyme-linked immunosorbent assay

EL4 cells were stimulated with PMA (12.5 ng/ml) /Iono (1 µM) for 16hr and culture supernatant was collected. The level of sγc was detected using a sandwich enzyme-linked immunosorbent assay with γc-specific polyclonal antibodies (R&D Systems, Minneapolis, MN, USA) as capture antibodies and biotin-conjugated γc-specific monoclonal antibodies (4G3; BD) as detection antibodies, as previously described [5, 6, 24]. Recombinant sγc protein was used as the positive control.

### Immunoblotting

Cell lysates were obtained from stimulated LN T, EL4, and EL4 KO, EL4 mutant cell lines, resolved using sodium dodecyl sulphate–polyacrylamide gel electrophoresis (12% acrylamide; Invitrogen) under reducing conditions, and transferred to polyvinylidene difluoride membranes (Amersham Biosciences, Amersham, Buckinghamshire, U.K). Blots were incubated with biotinylated anti-mouse IL-2Rγ (1:200, R&D system), p65 (F-6), cRel (C), NFATc2 (4G6-G5) (1:1000, all from Santa Cruz Biotechnology), and GAPDH, phospho p65(ser536), phospho AKT (ser473), phospho MEK1/2 (Ser217/221) (1:1000, Cell Signaling, Danvers, Massachusetts, USA) antibodies, followed by horseradish peroxidase (HRP)-conjugated streptavidin (1:2000, BioLegend) or HRP-conjugated anti-rabbit or anti-mouse IgG (1:5000, Cell Signaling) and HRP-conjugated anti-mouse β-actin antibodies (1:2000, Santa Cruz Biotechnology). The membranes were then incubated with enhanced chemiluminescence to determine the reagents (Amersham™, Amersham, Buckinghamshire, U.K) and exposed using the LAS-3000 Imaging system (Fujifilm, Minato-ku, Tokyo, Japan) and Imager 680 (Amersham™). The summary graph indicates the densitometric analysis of western blots (relative intensity) with an arbitrary number of the intensity ratio of γc to β-actin or GAPDH using ImageJ software.

### DNA constructs and luciferase promoter assay

The deletion versions of γc promoter regions were produced by serially eliminating the predicted NFAT1 binding sites and were inserted into the pGL4 reporter vector using XhoI and HindIII restriction enzymes (New England Biolabs). To generate NFAT1-binding site-deleted constructs, the different forward primers and equal reverse primers in the first exon of the γc gene were used. The primer sequences used to obtain different combinations of the construct were as follows: forward, 1397 bp, 5'-CACTAACACTCTCTCCCCCAGA-3'; 910 bp, 5'-CCAGTTTGTGGGTTACGGGA-3'; 743 bp, 5'-TGAGGTTTCAAGTCGGGCAG-3'; 513 bp, 5'-GTACCCAC ATGAATCATGTCAG-3'; 278 bp, 5'-TCTCCCTGGGGACTTAGCTT-3'; 194 bp, 5'-CCGGAAGCTACGACAAAAGG-3'; 167 bp, 5'-GGAGAGTGGTTCAGGGTTCT-3'; and reverse, 5'-TTCGCACTGGACATGAGGAC-3'. The sequences of all the constructs were confirmed using sequencing. To generate NFκB-binding site-deleted constructs, different forward primers and equal reverse primers in the first exon of the γc gene were used. The primer sequences used to obtain different combinations of the construct were as follows: forward, 1397 bp, 5'- CACTAACACTCTCTCCCC CAGA-3'; 910 bp, 5'- CCAGTTTGTGGGTTACGGGA-3'; 672 bp, 5 '-TTTGCAGGGAGCTAGGAAGT-3'; 514 bp, 5'-AGTACCCACATGAATCA-3 '; 278 bp, 5'-TCTCCCTGGGGACTTAGCTT-3'; 167 bp, 5'- GGAGAGTGGTTCAGGGTTCT -3', and reverse, 5'-TTCGCACTGGACATGAGGAC-3'. The sequences of all the constructs were confirmed using sequencing. The two NFAT1-binding sites at positions -737 and -111 were subjected to site-directed mutagenesis (-737 site: GGAA to mutant GTCA; -111 site: TTCC to mutant TGAC). The three NFκB-binding sites at positions -440, -180, and -114 were subjected to site-directed mutagenesis (-440 site; GGAA to mutant TTAA; -180 site; GGGA to mutant TTAA; -114 site; GGTT to mutant TTTT). The sequences of the cloned DNA fragments were confirmed using DNA sequencing.

The human embryonic kidney cell line HEK293 T, received from Dr. Sang-Mo Kwon (Pusan National University, South Korea), was transiently co-transfected with full-length, NFAT1, or NFκB-binding site-deleted γc promoter constructs containing a reporter vector, together with NFAT1 or NFκB-expressing vectors using Lipofectamine™ 2000 (Invitrogen) for 16 h. The hRluc vector, which expresses *Renilla* luciferase activity, was also transfected to normalize the firefly luciferase activity of the pGL4 vector. After 16 hr, the HEK293 T cells were stimulated with PMA/Iono for 6 h and then lysed using a dual-luciferase assay system (Promega, Madison, WI, USA). Firefly and *Renilla* luciferase activities were measured using a Victor 3 multi-well plate reader (Perkin Elmer, Waltham, MA, USA).

### Chromatin immunoprecipitation (ChIP) assay

T cells were stimulated with α-TCR/α-CD28 for 16 hr, crosslinked for 10 min at 37 °C using 1% formaldehyde, and lysed. The crosslinking reaction was arrested by exposure to glycine in phosphate-buffered saline, and the cell layer was collected in phosphate-buffered saline containing protease and phosphatase inhibitors. The chromatin was sheared by sonication on ice. Lysates were incubated with antibodies against NFAT1 and NFκB (p65 and cRel), or without antibodies, overnight at 4 °C, and then, protein was digested using proteinase K (0.4 mg/ml). The ChIP-enriched DNA was subjected to qPCR. Each experiment was performed two or three independent times. ChIP and non-antibody signals were normalized to the total input. The primer pairs used for detecting γc promoter were NFAT1 #1 (forward, 5'-AACACTCTCTCCCCCAGAAAA-3'; reverse, 5'-AACAAAGGTAGGAACCAGCCA-3'), NFAT1 #2 (forward, 5-CCAGTTTGTGGGTTACGGGA-3'; reverse, 5'-CAGCCCTGTTTCTGCGGTAT-3'), NFAT1 #3 (forward, 5'-AGCAGTTAGGGGTGGCTA TTC-3'; reverse, 5'-CACCACACACCATCATTCCC-3'), NFAT1 #4 (forward, 5'-CATCACCTTAGAGCAGAACCCA-3'; reverse, 5'-GAACCCTGAACCACTCTCCC-3'), NFκB #1 (forward, 5'-AAGCACTGTACCGAGCACAT-3'; reverse, 5'-TCCCGTAACCCACAAACTGG-3'), NFκB #2 (forward, 5'-AAGCACTGTACCGAGCACAT-3'; reverse, 5'-GACT TCCTAGCTCCCTGCAA-3'), NFκB #3 (forward, 5'-AAGCACTGTACCGAGCACAT-3'; reverse, 5'-TGGGTTCTGCTCTAAGGTGATG-3'), and NFκB #4 (forward, 5'-AGGGTCCTGAAGGGTCTT GA-3'; reverse, 5'-GAACCCTGAACCACTCTCCC-3').

### Generation of γc promoter-mutant EL4 cell line using the CRISPR/Cas9 system

To generate γc promoter -111 (TC→GA) mutant cells, 1.5 × 10^5^ EL4 cells were collected and resuspended in electroporation solution (Buffer R), and ssODNs (200 pmol, Macrogen, Soeul, Republic of Korea) and Cas9 (100 pmol, Enzynomics)/sgRNA (250 pmol, IDT, Coralville, IA, USA) were added to the resuspended cells. Electroporation was conducted using a Neon Transfection System (Invitrogen). Electroporated cells were slowly loaded into a growth medium with RS-1 (22.5 µM; Sigma-Aldrich) and cultured at 37 °C under 5% CO_2_ conditions; 48 hr after transfection, genomic DNA was purified using Proteinase K extraction buffer. The indel ratio, homology-directed repair frequency, and genomic DNA patterns were analyzed using Illumina Mini-Seq and Cas-Analyzer, as mentioned above [21].

### Statistical analysis

The data are shown as the mean ± SEM. Statistical differences between groups were examined using Student’s two-tailed* t*-tests or one-way ANOVA with GraphPad Prism (GraphPad Software). *p* values of < 0.05 were considered statistically significant. *p < 0.05, ** p < 0.01, *** *p* < 0.001, **** *p* < 0.0001.

## Results

### Upregulation of γc expression in activated T cells

**Fig. 1:**
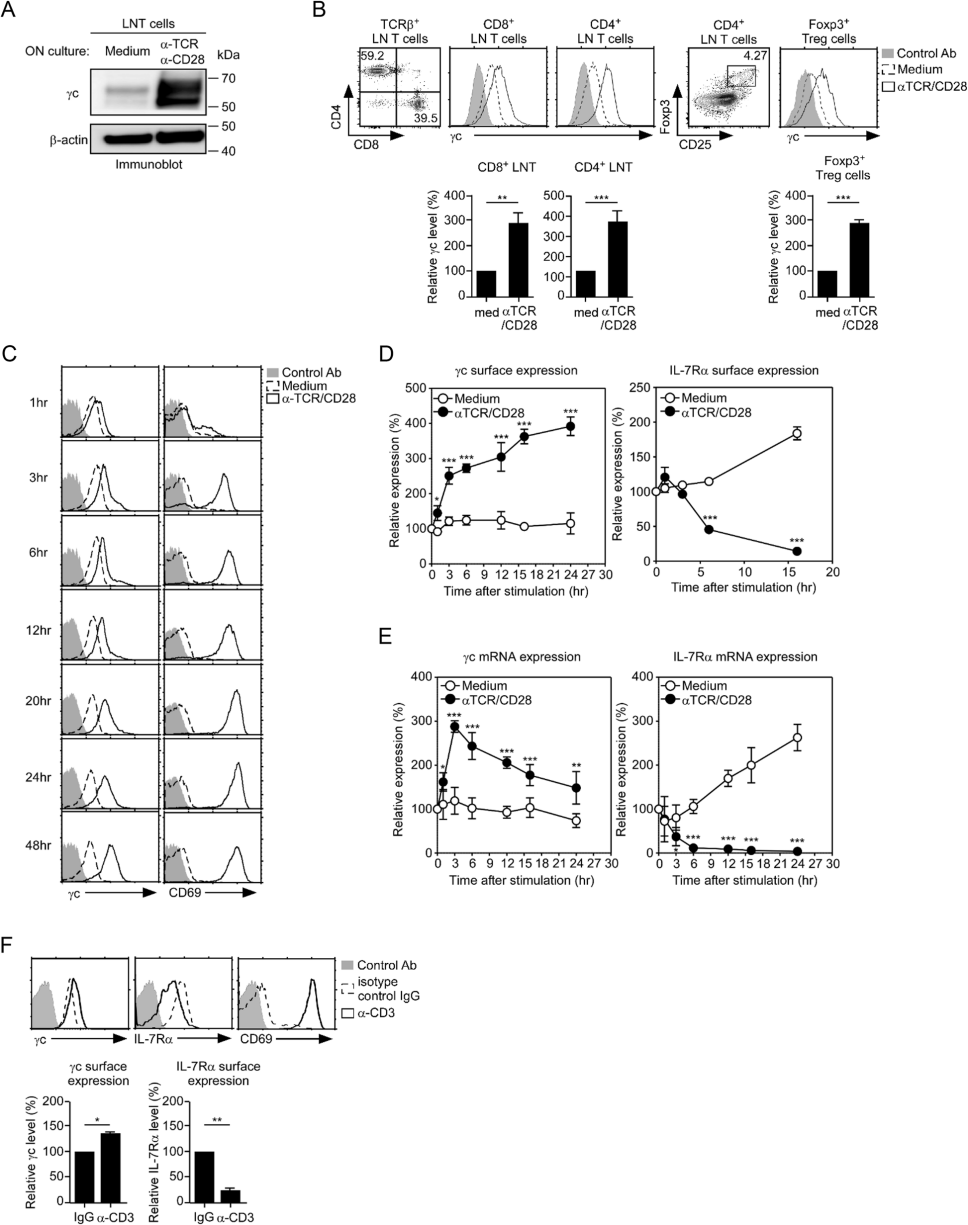
Upregulation of γc expression in LN T cells upon TCR stimulation. **A** Immunoblot detection of γc proteins in activated T cells. T cells were stimulated with α-TCR (1 µg/ml) / α-CD28 (1 µg/ml) for 16 h. β-actin was the loading control. The results are the summary of three independent experiments. **B** Surface γc expression in each T cell subset including CD4^+^, CD8^+^, and regulatory T cells upon TCR stimulation. LN T cells were stimulated with α-TCR/α-CD28, and the γc expression was analyzed using FACS analysis. γc expression levels (open histogram) were assessed in activated CD8^+^ T cells, CD4^+^ T cells, and CD25^+^Foxp3^+^ Treg cells and overlaid with staining control antibody (shaded histogram). Bar graphs are the summary of two independent experiments (three mice per group). **C** Time-dependent kinetics of γc expression with TCR stimulation. LN CD8^+^ T cells were stimulated with α-TCR/α-CD28, and the cytokine receptor and activation marker expression was analyzed using FACS at the indicated time points. CD69 and γc expression levels (open histogram) were assessed in activated LN CD8^+^ T cells and overlaid with control antibody staining (shaded histogram). **D** γc and IL-7Rα expression in activated CD8^+^ T cells were determined using FACS at the indicated time points. The results are the summary of three independent experiments. **E** Relative *γc* and *IL-7Rα* mRNA expression were determined using RT-qPCR and normalized to expression in medium, which was set to 100%. The results are the summary of three independent experiments. **F** (Top) γc, CD69, and IL-7Rα expression in BALB/c mice injected overnight with 10 μg α-CD3 antibody. (Bottom) Relative γc and IL-7Rα surface expression determined using FACS and normalized to expression in isotype control IgG used as a control for the specific α-CD3 antibody for in vivo T cell stimulation, which was set to 100%. The results are the summary of three independent experiments (three mice per group). Data are presented as the mean ± standard error of the mean of three independent experiments. * p < 0.05, ** p < 0.01, and *** p < 0.001

As we previously reported, sγc expression is dynamically upregulated upon TCR stimulation [5]. Therefore, we determined whether membrane γc (mγc) expression is regulated by T cell stimulation. First, we stimulated LN T cells with antibodies against α-TCR and α-CD28 for overnight and then examined the γc expression level. The total amount of γc increased significantly upon TCR stimulation (Fig. 1A). Next, γc expression in different subset of T cells including CD4^+^, CD8^+^ and regulatory T cells was assessed upon TCR stimulation. We found that increase of γc expression upon TCR stimulation was consistent across these different T cell subsets (Fig. 1B). γc surface expression rapidly increased and was upregulated in a time-dependent manner upon TCR stimulation. The enhanced γc surface expression was maintained for 48 h (Fig. 1C and D), and the expression of CD69 an activation marker was upregulated (Fig. 1C). Although IL-7Rα expression was significantly downregulated by TCR stimulation, γc surface expression was significantly upregulated in activated T cells (Fig. 1D). γc mRNA expression correlated with γc surface expression during the early time point of TCR signals, whereas IL-7Rα surface expression correlated with the mRNA expression in a time-dependent manner (Fig. 1E). TCR signaling transiently upregulated γc mRNA expression; however, its levels recovered to those observed in resting T cells (Fig. 1E). Moreover, we assessed mγc levels in BALB/c mice injected with α-CD3 antibodies to induce acute polyclonal T cell stimulation in vivo. We found that the mγc levels significantly increased after overnight α-CD3 antibody injection, concomitant with T cell activation, as evident by the expression of surface IL-7Rα and CD69 (Fig. 1F). These results demonstrated that γc level was actively upregulated during T cell activation both in vitro and in vivo.

### Transcriptional regulation of γc expression in LN T cells via TCR signaling

**Fig. 2:**
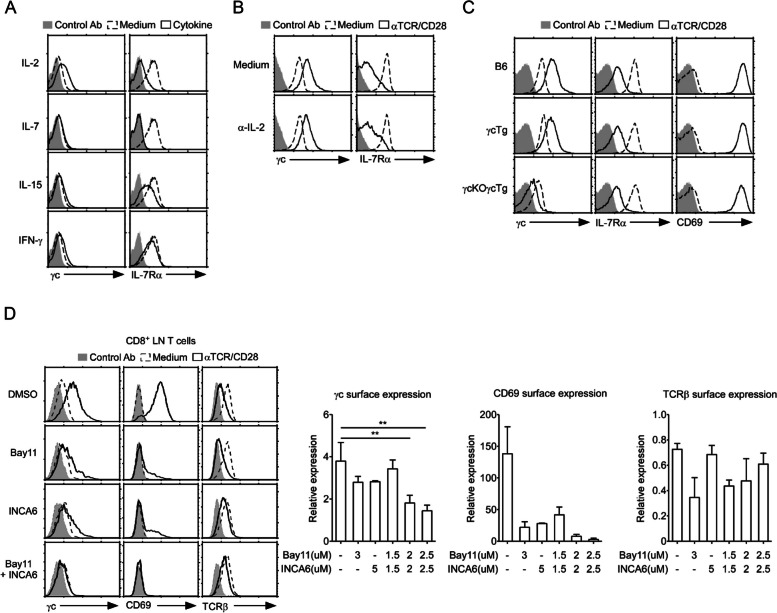
Transcriptional control of γc expression via TCR signaling. **A** Upregulation of surface γc expression by IL-2. CD8^+^ T cells were stimulated with γc cytokines (10 ng/ml IL-2, 10 ng/ml IL-7, and 100 ng/ml IL-15) or non-γc cytokine (10 ng/ml IFN-γ) for 16 h, and γc and IL-7Rα expression was analyzed using FACS. Data are representative of three independent experiments. **B** Upregulated expression of surface γc partially blocked by 10 µg/ml α-IL-2. CD8^+^ T cells were stimulated with α-TCR/α-CD28 in the absence or presence of α‐IL‐2. Surface expression of IL-7Rα and γc was determined using FACS. The results are the summary of three independent experiments. **C** Removal of transcriptional regulation completely suppresses upregulation of γc expression. CD8^+^ T cells from B6, γcTg, and γcKOγcTg mice were assessed for γc surface (left), IL-7Rα (middle), and CD69 (right). γc, IL-7Rα, and CD69 expression (open histogram) versus control antibody staining (shaded histogram) are shown for CD8^+^ T cells from B6, γcTg, and γcKOγcTg mice. The results are the summary of three independent experiments. **D** CD8^+^ T cells were stimulated with α-TCR/α-CD28 in the presence or absence of Bay11 and INCA6 as specific inhibitors for NFκB and NFAT1, respectively. γc (left), CD69 (middle), and TCRβ (right) surface expression was analyzed using FACS. Histograms show the representative data from four independent experiments. The bar graph shows the relative surface expression of γc, CD69, and TCRβ. The relative γc, CD69, and TCRβ expression was calculated as the Δ mean fluorescence intensity (ΔMFI) associated with the inhibitor-treated group over the ΔMFI associated with the DMSO control group. DMSO was used as vehicle control. Data are presented as the mean ± standard error of the mean of three independent experiments. ** p < 0.01

TCR activation promoted several signaling cascades that regulate cytokine production and cell survival, proliferation, and differentiation. For example, TCR stimulation induced IL-2 production, and IL-2 signaling upregulated the expression of IL-2Rα. To determine whether γc expression was upregulated by indirect alterations, such as upregulated IL-2 expression by TCR activation, we stimulated LN T cells with γc family cytokines (IL-2, IL-7, and IL-15) and IFN-γ. Unlike the effect of IL-7 and IL-15 stimulation, which was not significant to γc expression, IL-2 slightly enhanced the expression level (Fig. 2A). Conversely, IL-7Rα expression was downregulated upon IL-2 stimulation (Fig. 2A). To further confirm whether IL-2 signaling was involved in the upregulation of γc expression upon TCR stimulation, we stimulated LN T cells in the presence of α-IL-2 to block IL-2 signaling. Although IL-2 signaling was blocked, γc expression was upregulated upon TCR stimulation (Fig. 2B). Collectively, these results indicated that γc expression was directly upregulated by TCR signaling. To further confirm if γc was transcriptionally regulated, we determined γc expression levels in γcTg and γcKOγcTg T cells after TCR stimulation. While γc surface expression in γcTg T cells was upregulated as in WT mice, γc expression in γcKOγcTg T cells was downregulated upon TCR stimulation. The changes in IL-7Rα and CD69 expression in γcKOγcTg mice were comparable to those in WT mice (Fig. 2C). To clarify which signaling pathway was involved in the regulation γc gene expression, we first stimulated T cells with α-TCR/α-CD28 in the presence of PD980 (an ERK pathway inhibitor) or wortmannin (an AKT pathway inhibitor) (Fig. S1A and B). The inhibition of the ERK and AKT pathway was evaluated by western blot analysis. Both inhibitors specifically inhibited these pathways in activated T cells (Fig. S1A). Regardless of the inhibition of the ERK or AKT pathway, γc expression was upregulated (Fig. S1B). NFκB and NFAT1 are representative TFs in the activation of the immune and inflammatory responses in T cells. Thus, to investigate whether these TFs control γc gene expression, we treated activated T cells with Bay11 or INCA6 to interrupt NFκB or NFAT1 signaling, respectively. Optimal concentration was selected based on cell viability, determined through assessment of inhibitors-induced cell death (Fig. S1C). Moreover, we confirmed the inhibition of NFAT1 and NFκB pathways by western blot analysis (Fig. S1D). The upregulation of γc expression was inhibited by treatment with Bay11 or INCA6 at both the mRNA and protein levels and was more critically blocked by a combination of Bay11 and INCA6 (Fig. 2D and S1E). Taken together, these results indicated that γc expression was transcriptionally controlled by NFκB and NFAT1 but not by the AKT or ERK signaling pathway.

### γc expression in NFAT1 and NFκB KO EL4 cells

**Fig. 3:**
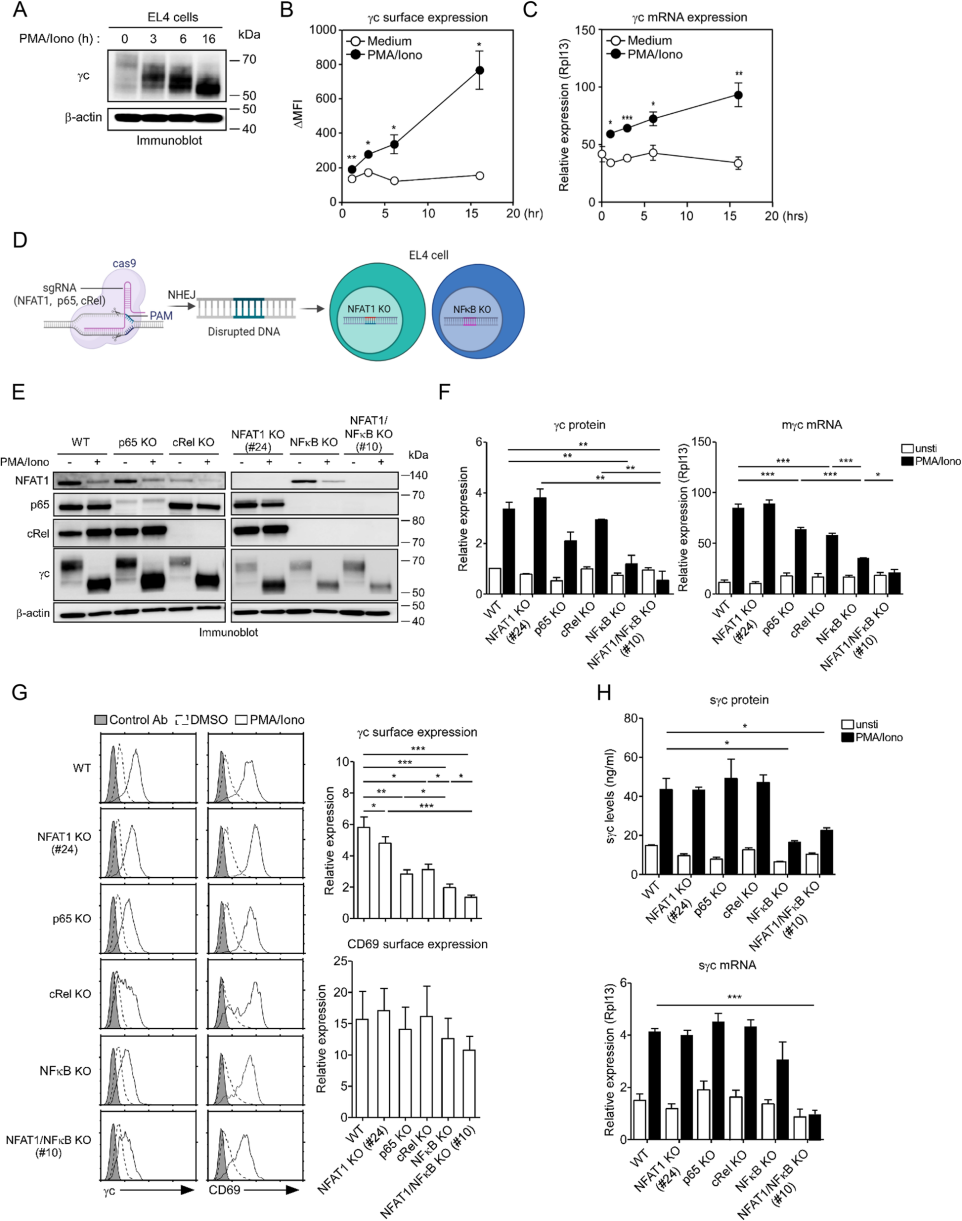
NFκB and NFAT1 are required to upregulate γc expression. **A** Expression of γc protein increases in a time-dependent manner upon PMA (12.5 ng/ml) /Iono (1 µM) stimulation. The blot is representative of four independent experiments. **B** Determination of γc surface expression level using FACS and (**C**) γc mRNA level using RT-qPCR. The relative gene expression was calculated with that of the *Rpl13* housekeeping gene. **D** Experimental schematic of the generation of NFAT1 and NFκB KO EL4 cell lines using CRISPR/Cas9. **E** Generation of NFAT1 and NFκB (p65 and cRel) KO EL4 cell lines using the CRISPR/Cas9 system. WT, NFAT1 KO (#24), p65 KO, cRel KO, NFκB KO, and NFAT1/NFκB KO (#10) EL4 cells were stimulated with PMA/Iono for 16 h. NFAT1, NFκB, and γc protein levels in the indicated cell lines were analyzed using western blotting. β-actin was used as the loading control. Data are representative of four independent experiments. **F** Immunoblot analysis of γc expression in the presence of PMA/Iono (left); determination of membrane γc (mγc) mRNA expression using RT-qPCR (right). The relative gene expression was calculated with that of the β-actin and *Rpl13* housekeeping genes. The results are the summary of four independent experiments. **G** γc expression in KO EL4 cell lines. KO EL4 cell lines were stimulated with PMA/Iono, and the cytokine receptor and activation marker expression was analyzed using FACS: (left) γc and CD69 expression (open histogram) assessed in activated KO EL4 cell lines and overlaid with control antibody staining (shaded histogram); (right) summary of FACS analysis for γc and CD69 expression in the presence of PMA/Iono. The results are the summary of four independent experiments. **H** sγc expression synergistically downregulated in NFκB KO and NFAT1/NFκB KO (#10) cells (Top). KO cells were stimulated with PMA/Iono and culture supernatants were harvested to assess soluble γc (sγc) expression. The results are the summary of four independent experiments. sγc mRNA expression was assessed using RT-qPCR (bottom). The relative gene expression was calculated with that of the *Rpl13* housekeeping gene. Data are presented as the mean ± standard error of the mean of four independent experiments. * p < 0.05, ** p < 0.01, and ***p < 0.001

To further confirm the role of NFκB and NFAT1 in the upregulation of γc expression in activated LN T cells, we tested loss-of-function of NFAT1 and NFκB (p65 and cRel) using EL4 cells, which are murine lymphoma T cells. Prior to this, we first examined whether γc expression in EL4 cells was regulated by TCR stimulation, similar to that in LN T cells. We found that γc expression was upregulated in a time-dependent manner at the protein and mRNA levels with stimulated PMA/Iono (Fig. 3A-C and S2A). Next, we investigated whether γc expression was regulated during the transcriptional process. EL4 cells were stimulated with PMA/Iono in the presence of DRB (an RNA polymerase II inhibitor) or CHX (a protein synthesis inhibitor). Upregulation of γc expression was significantly inhibited by both DRB and CHX, and CD69 expression was downregulated by CHX but not by DRB in EL4 cells (Fig. S2B). Consequently, γc expression levels were transcriptionally regulated. To reconfirm whether γc expression was regulated through the NFAT1 or NFκB pathway in EL4 cells, we stimulated EL4 cells in the presence or absence of NFAT1 (CsA or INCA6) and NFκB (MG132 or Bay11) inhibitors alone and in combination. The upregulation of γc protein and mRNA expression was significantly inhibited in the presence of MG132 or CsA (Fig. S2C and S2D). Similarly, the upregulation of γc expression was inhibited by treatment with Bay11 or INCA6 at both the mRNA and protein levels and was more synergistically blocked by a combination of Bay11 and INCA6 (Fig. S2E). NFAT1 and NFκB inhibitors suppressed the upregulation of γc expression in activated T cells. As the regulatory mechanism of γc expression was similarly confirmed in EL4 cells to LN T cells, we generated the NFAT1 and NFκB (p65 and cRel) KO EL4 cell lines using CRISPR/Cas9 (Fig. 3D) and obtained distinct clones 2 NFAT1 KO clones (#21 and #24) and 2 NFAT1/NFκB KO clones (#7 and #10). First, we confirmed that the expression of NFAT1 and NFκB was knocked out at the protein and mRNA levels (Fig. S3A, S3C-D). The KO cells stimulated with PMA/Iono failed to significantly upregulate γc expression (Fig. 3E and F) and the results were consistent across different clones of each KO cell line (Fig. S3A and S3B); in particular, the upregulation of γc expression in NFκB KO and NFAT1/NFκB KO cells was synergistically inhibited at the mRNA and protein levels (Fig. 3F and G). CD69 surface expression in WT EL4 and each KO EL4 cell was comparable (Fig. 3G). The mRNA and protein levels of sγc were decreased in NFκB KO and NFAT1/NFκB KO cells (Fig. 3H). Taken together, the expression of both membrane and soluble γc was significantly suppressed in NFAT1/NFκB KO cells. These results suggested that both NFAT1 and NFκB are required for optimal regulation of γc expression in activated T cells.

### Functional cooperation and physical binding of NFAT1 and NFκB to the γc promoter locus

**Fig. 4:**
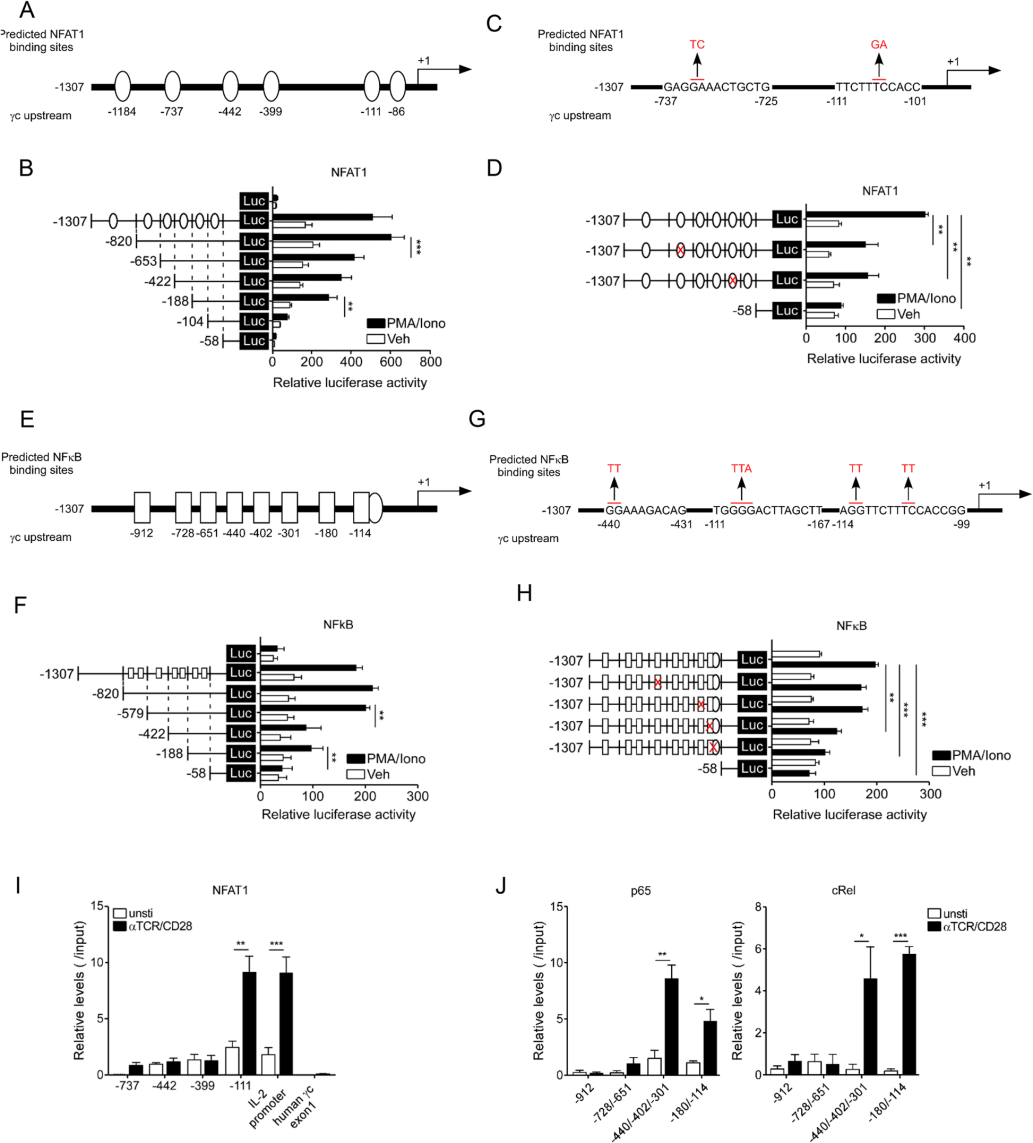
In vitro and in vivo binding of NFAT1 and NFκB to the γc promoter locus. **A** Schematic of the six predicted NFAT1-γc promoter region binding sites. **B** HEK293 T cells were transfected with control pGL4-luc or each of the pGL4-NFAT1-luc plasmid that carried -1307, -820, -653, -422, -188, and -104 of the γc promoter region and were then stimulated with PMA/Iono for 6 hr. Luciferase activity in the lysates of transfected cells was determined by a dual luciferase assay. The results are the summary of three independent experiments. **C** Schematic of pGL4-NFAT1-luc that carried point mutation at the -737 (GA to TC) and -111 (TC to GA) sites on the γc promoter. **D** The NFAT1 promoter activity in site mutation-transfected cells was measured by a dual luciferase assay. The results are the summary of three independent experiments. **E** Schematic of eight predicted NFκB-γc upstream region binding sites. **F** HEK293 T cells were transfected with each of the pGL4-NFκB-luc plasmid that carried -1307, -820, -579, -422, and -188 of the γc promoter region and were then stimulated with PMA/Iono for 6 hr. Luciferase activity in the lysates of transfected cells was determined by a dual luciferase assay. The results are the summary of three independent experiments. **G** Schematic of pGL4- NFκB-luc that carried point mutations at the -440 (GG to TT), -180 (GGG to TTA), and -114 (GG to TT and TC to TT) sites on the γc promoter. **H** The NFκB promoter activity in site mutant cells was determined by a dual luciferase assay. The results are the summary of three independent experiments. **I** and **J** Chromatin from LN T cells stimulated with α-TCR/α-CD28 was analyzed for recruitment of NFAT1 or NFκB (p65 and cRel) to the γc promoter by ChIP (as described in the Materials and Methods). IL-2 promoter and human γc exon1 were used as positive and negative controls for the ChIP assay, respectively. The quantity of DNA in the precipitation with NFAT1, p65, and cRel antibodies was normalized to input chromatin and plotted relative to the IgG background. Data are presented as the mean ± standard error of the mean of four independent experiments. * p < 0.05, ** p < 0.01, and *** p < 0.001

**Fig. 5:**
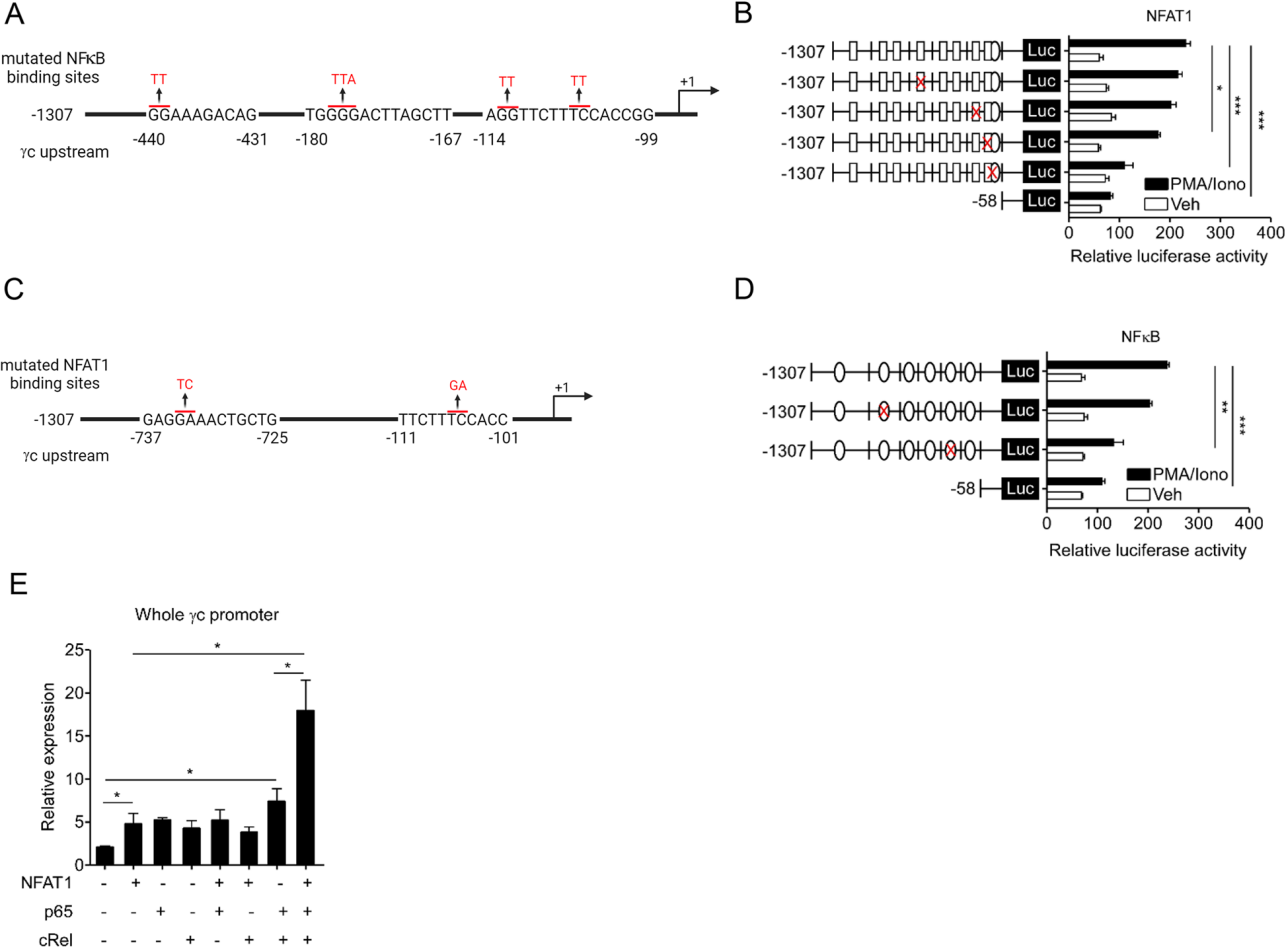
NFAT1 and NFκB cooperatively activate the γc promoter. **A** Schematic of the mutated NFκB-binding sites -440 (GG to TT), -180 (GGG to TTA), and -114 (GG to TT and TC to TT) regions on the γc promoter. **B** HEK293 T cells were transfected with control pGL4-luc or each of the pGL4-NFAT1-luc plasmids that carried point mutation at the -440, -180, and -114 sites of the γc promoter region and were stimulated with PMA/Iono for 6 hr. The results are the summary of three independent experiments. **C** Schematic of mutated NFAT1-binding sites of the -737 (GA to TC) and -111 (TC to GA) region on the γc promoter. **D** HEK293 T cells were transfected with control pGL4-luc or each of the pGL4-NFκB-luc plasmids that carried point mutations at the -737 and -111 sites of the γc promoter region and were then stimulated with PMA/Iono for 6 hr. The results are the summary of three independent experiments. **E** HEK293 T cells were transfected with the indicated plasmids that carried the entire γc promoter region and were then stimulated with PMA/Iono for 6 hr. Luciferase activity in the lysates of transfected cells was determined by a dual luciferase assay. Data are presented as the mean ± standard error of the mean of four independent experiments. * p < 0.05, ** p < 0.01, and *** p < 0.001

To predict the potential regulatory elements in the upstream region of the γc gene, several potential regulatory elements that have highly conserved binding sites for NFAT1 and NFκB between humans and mice were determined using rVISTA 2.0 and TRANSFAC database analysis (Figure S4A and B). We identified six NFAT1- (Fig. 4A) and eight NFκB-binding motifs (Fig. 4E). To identify the key cis-regulatory elements, we generated a series of deletion constructs (Fig. 4A) and found that promoter activity was reduced in the -653/ + 91 and -104/ + 91 regions, implying that the -737 and -111 regions may have an NFAT1-binding motif (Fig. 4B). Furthermore, the functional sites for NFAT1 binding were mutated at positions -737 or -111, as shown in Fig. 4C. Mutation of the -734 to -733 (GA→TC) or -106 to -105 (TC→GA) sites significantly decreased the γc promoter activity, which confirmed the importance of these two sites (Fig. 4D). To identify critical NFκB-binding sites on the γc promoter, serially deleted constructs were generated, and promoter activity was assessed with these constructs (Fig. 4E). Deletion of the functional promoter region significantly decreased promoter activity, as shown in the -422/ + 91 and -58/ + 91 regions (Fig. 4F). Furthermore, we mutated three NFκB-binding sites: -440 to -439 (GG→TT), -177 to -175 (GGG→TTA), and -113 to -112 (GG→TT) or -106 to -105 (TC→GA, NFAT1- and NFκB-binding sites) and accessed their promoter activity upon PMA/Iono stimulation (Fig. 4G). Two mutated binding sites, -177 to -175 and -113 to -112, decreased γc promoter activity (Fig. 4H). Subsequently, to detect the presence of any physical binding of NFAT1 to the identified NFAT1- and NFκB-binding sites on the γc promoter, we conducted a ChIP assay using antibodies against NFAT1 and NFκB (p65 and cRel) in LN T cells stimulated with α-TCR/α-CD28. IL-2 promoter and human γc exon1 were used as positive and negative controls for the ChIP assay, respectively. In vivo binding of NFAT1 to the -111 region and of NFκB to the -440/-402/-301 and -180/-114 regions was confirmed in T cells, and their binding to the loci was further enhanced by TCR stimulation (Fig. 4I and J). In addition, the effects of the NFAT1 function of the NFκB-binding site mutant constructs (Fig. 5A) and the NFκB function of the NFAT1-binding site mutant constructs (Fig. 5C) were assessed for γc promoter activity via a luciferase assay. γc promoter activity was significantly reduced in the cross-binding sites mutation -114 (NFκB) and -111 (NFAT1) construction (Fig. 5 B‒D). The reduced γc promoter activity may have been due to the presence of overlapping (adjacent) binding sites for NFκB (-111 to -101) and NFAT1 (-114 to -99) on the γc promoter. A γc promoter-driven luciferase construct was transfected with NFAT1 or NFκB (p65 or cRel) or in combination with NFAT1/NFκB, and transactivity was measured via a luciferase assay. We found that promoter activity was increased for NFAT1 or NFκB (p65 and cRel) compared to that for the control (“Mock”) and was more synergistically induced with both NFAT1/NFκB (Fig. 5E). Taken together, these results indicated that the cooperation of NFκB and NFAT1 induced optimal expression of the γc protein in activated T cells, resulting from synergistic activity on the γc promoter.

### Regulation of γc expression in γc promoter mutant cells

**Fig. 6:**
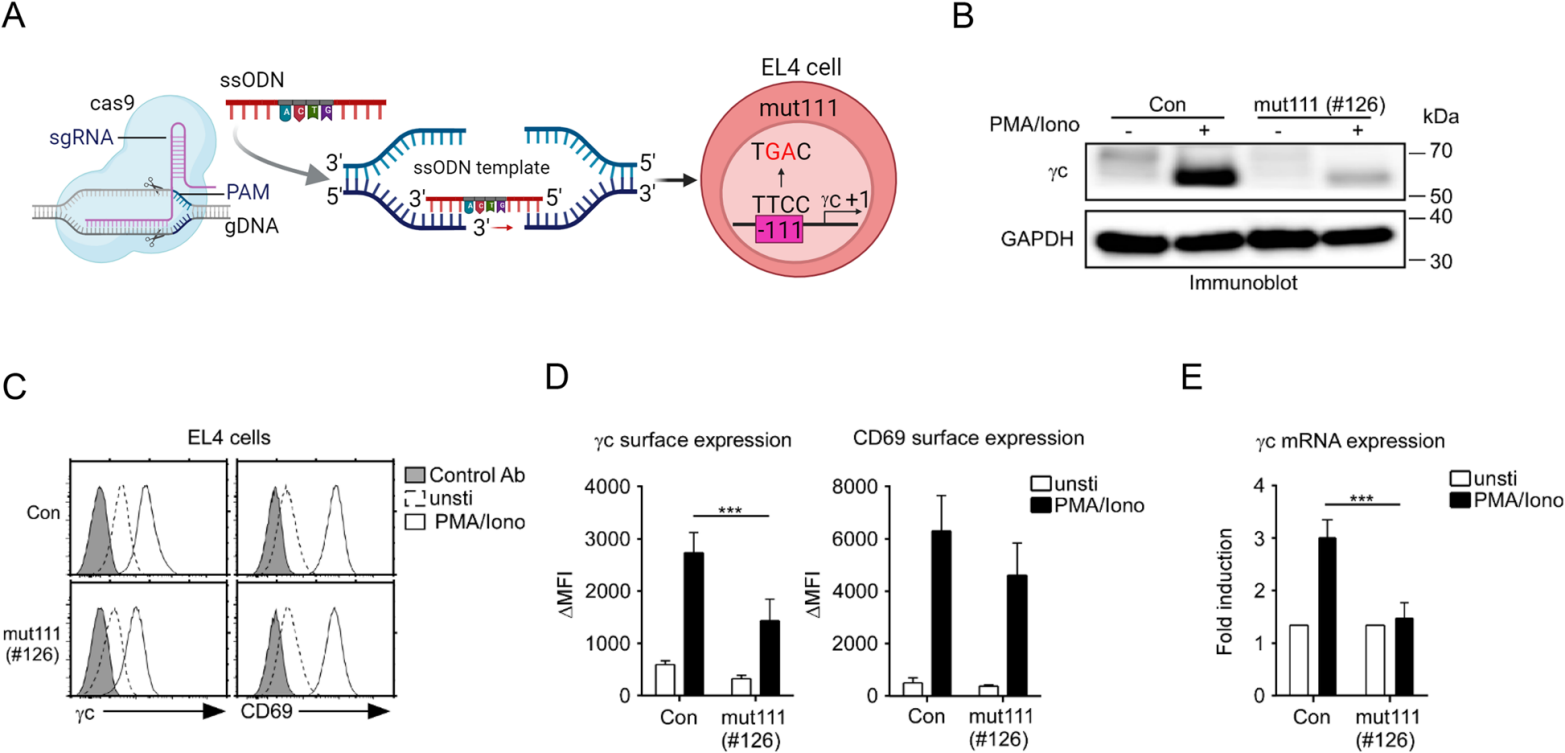
Key regulatory site involved in γc expression. **A** Generation of mutant EL4 cell line (NFAT1/NFκB-binding site on the γc promoter region; mut111) using the CRISPR/Cas9 system. **B** Western blot analysis of γc expression in mut111 (#126) and control EL4 cells. GAPDH was used as the loading control. Blot is representative of three independent experiments. **C** The mut111 (#126) cells were stimulated with PMA/Iono for 16 hr, and γc and CD69 were analyzed using FACS. γc and CD69 expression (open histogram) were overlaid with control antibody staining (shaded histogram). **D** Summary of FACS analysis for γc and CD69 expression in the presence of PMA/Iono. The results are the summary of five independent experiments. **E** mRNA expression of γc in mut111 (#126) and control EL4 cells was determined using RT-qPCR. The relative gene expression was calculated with that of the *Rpl13* housekeeping gene. The results are the summary of five independent experiments. *** *p* < 0.001

To further confirm the vital binding sites of NFAT1 and NFκΒ, the -106 to -105 region of the γc promoter in EL4 cells was mutated from TC to GA (mut111) using CRISPR/Cas9-mediated gene editing (Fig. 6A). We obtained homozygous mutant clones (mut111; #120 and #126) and confirmed their mutation with NGS-seq analysis. We stimulated mut111 (#120 and #126) clones with PMA/Iono and found that the total amount of γc protein was efficiently suppressed in mut111 (#120 and #126) clones; in contrast, the expression of γc protein in the control was effectively enhanced by PMA/Iono (Fig. 6B and Fig. S5A), and surface γc expression was not significantly upregulated compared to that in control cells (Fig. 6C, D and Fig. S5B). CD69 expression was comparable between the mut11 (#120 and #126) clones and control groups (Fig. 6C and Fig. S5B). Similar to the protein levels, the mRNA levels of γc were not upregulated compared to those in the control cells (Fig. 6E). Taken together, these results indicated that the -111 to -101 region of the γc promoter was a key regulatory region of γc expression in activated T cells.

### Upregulation of γc expression inhibits IL-7 signaling

**Fig. 7:**
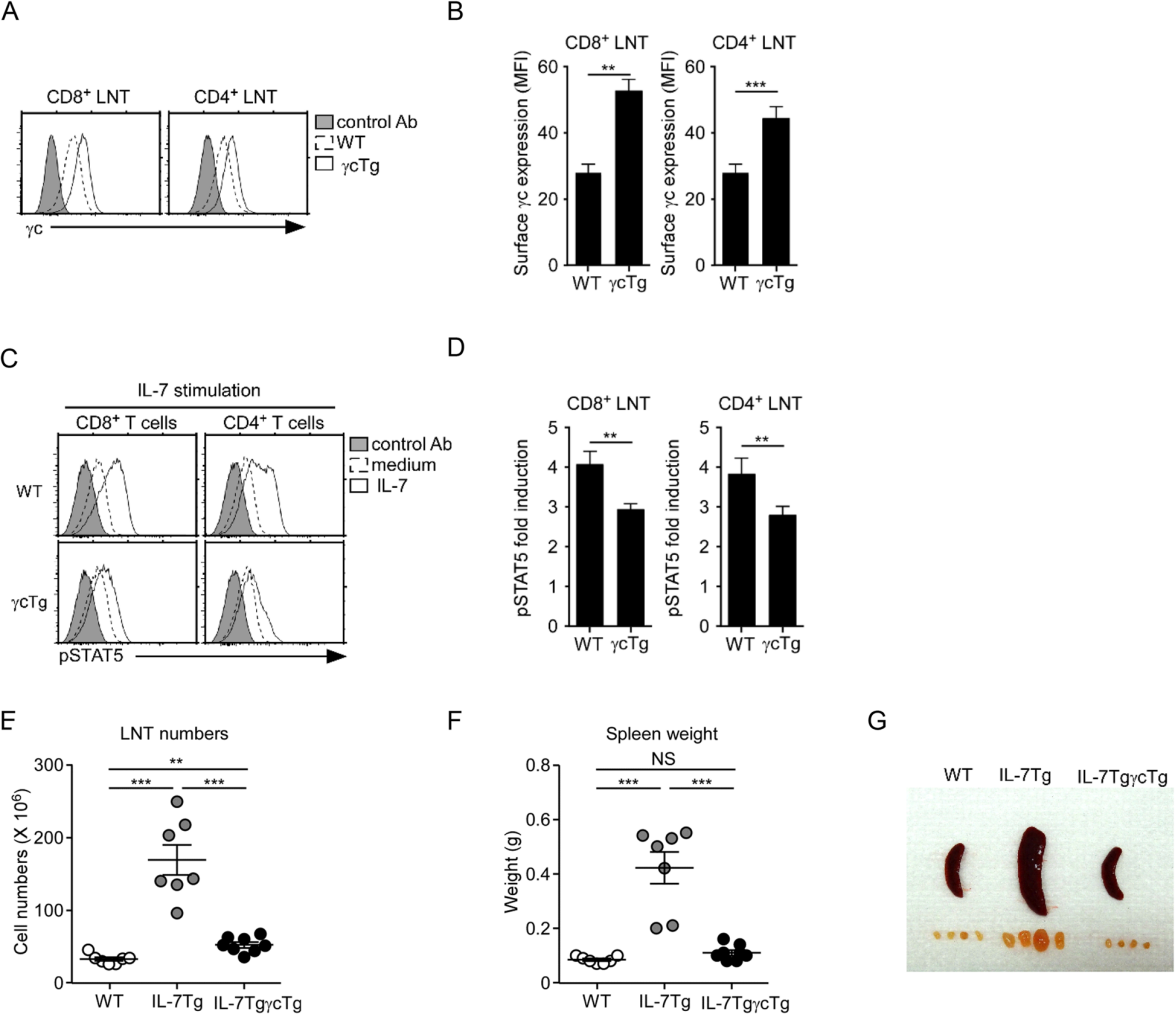
γc overexpression inhibits IL-7 signaling. **A** and **B** Surface γc expression in WT and γcTg CD4^+^ and CD8^+^ T cells (open histogram) as overlaid with control antibody (shaded histogram) staining: (**A**) the γc expression level was determined by flow cytometry, and (**B**) summary of the γc expression level data. **C** and **D** IL-7 downstream signaling in γc-overexpressing CD8^+^ and CD4^+^ T cells: (**C**) IL-7-stimulated LN T cells from WT and γcTg mice were assessed for pSTAT5 expression using flow cytometry, and (**D**) bar graphs show fold induction of IL-7-induced STAT5 phosphorylation over IL-7-untreated cells. Data are representative of eight independent experiments. **E** LN T cell numbers determined in IL-7Tg and IL-7TgγcTg mice. Each symbol represents an individual mouse. Horizontal lines indicate the mean ± standard error of the mean. **F** Spleen weight of IL-7Tg and IL-7TgγcTg mice. Each symbol represents an individual mouse. **G** Gross anatomy of spleens and LNs from the indicated mice. Image shows representative spleen and lymph nodes from > 5 mice per group. NS, non-significant; * p < 0.05, ** p < 0.01, and *** p < 0.001

Next, we investigated the reason as to why γc expression was upregulated after TCR stimulation. To address this, we generated γcTg mice in which the surface γc levels were significantly upregulated in both CD4^+^ and CD8^+^ T cells (Fig. 7 A and B). To assess any γc effects on T cells, we stimulated freshly isolated γcTg LN T cells with IL-7 and assessed STAT5 phosphorylation (Fig. 7C). pSTAT5 induction was suppressed in γc-overexpressing T cells (Fig. 7D). To further confirm γc effects in vivo, we crossed γcTg mice with an IL-7-overexpressing animal model that displayed B and T lymphoproliferation, resulting in lymphoma. T cell numbers (Fig. 7E) and spleen weights (Fig. 7F) were significantly increased in IL-7Tg mice; however, γc overexpression restored the number and weight of WT controls (Fig. 7G).

## Discussion

We aimed to identify the regulatory mechanisms controlling γc expression in activated T cells. Although γc expression is thought to be consistent in response to γc family cytokine signaling [1], the regulatory mechanism of γc expression has not previously been reported. We previously found that sγc expression is significantly upregulated in activated T cells [5, 6]. Thus, we hypothesized that γc expression is regulated in activated T cells. In this study, we present evidence that the NFAT1 and NFκB signaling pathways directly upregulate γc expression upon TCR stimulation of T cells. Although IL-2 signaling induced by TCR engagement moderately contributed to the upregulation of γc expression, TCR signaling was directly and predominantly involved in the upregulation of γc expression.

The initial TCR signaling response in naïve T cells enhances the expression of IL-2 and IL-2Rα [25]. The IL-2 receptor comprises IL-2Rα, β, and γ chains. Prior to activation, naïve T cells do not express IL-2Rα; however, IL-2Rα expression is upregulated in activated T cells [26]. IL-2Rα expression is regulated by various TFs and regulatory elements, including STAT5, NFAT, NFκB, AP-1, SP1, EGR1, and E74-like factor 1 (ELF1). IL-2Rβ expression is disparate in naïve CD8^+^ and CD4^+^ T cells, as evident by the substantial IL-2Rβ expression in naïve CD8^+^ T cells, which is remarkably impaired in naïve CD4^+^ T cells [22]. The regulatory mechanism of IL-2Rβ expression has been extensively investigated [22, 27], and multiple TFs, including Ets-1, T-bet, eomesodermin, GABP, SP1, and EGR1 [28], have been found to induce its expression on T cells. As the other γc expression is thought to be constitutively expressed on T cells and does not alter their expression, only limited information is available regarding TFs and the regulatory mechanism of γc expression. Although the γc promoter contains ETS-binding sites capable of interacting with GABP and ELF1 [29, 30], whether it plays a role in controlling γc expression remains unclear.

We first demonstrated a direct correlation between the upregulation of total γc expression and the induction of its mRNA expression, implying that TCR signaling directly regulates γc expression at the transcriptional level. We confirmed that γc expression was transcriptionally upregulated in activated T cells and validated the effect of the key TFs NFAT1 and NFκB on the upregulation in a CRISPR/Cas9 genome-edited cell line. Our study provides important information for future studies aimed at revealing the regulatory mechanisms of γc expression.

NFAT is one of the major TFs in T cells activated by TCR stimulation [9]. NFAT1 is mainly present in immune cells, such as T, B, and natural killer (NK) cells, and is rapidly dephosphorylated and translocated to the nucleus upon TCR stimulation [9]. Thus, we focused on NFAT1 as a TF involved in regulating γ*c* gene expression. The upregulation of γc expression was suppressed in a time-dependent manner by NFAT-specific inhibitors and NFAT1 knockdown and knockout. Exogenous NFAT1 enhanced γc promoter activity, which was correlated with γc surface expression levels. Furthermore, we identified critical NFAT1 binding sites at the -111 regions, which regulate γ*c* gene expression. Collectively, we found that dephosphorylated NFAT1 in activated T cells was translocated to the nucleus and bound to the -111 region of the γc promoter, resulting in positive regulation of γ*c* gene expression. NFAT1 proteins generally require partner TFs to positively regulate target genes in the nucleus [31]. We predicted NFAT1- and AP-1-binding sites on the γc promoter region. AP-1 family TFs, including Jun, Fos, ATF, and Maf subfamilies, constitute quaternary with NFAT1 proteins and DNA and consequently lead to target gene expression upon TCR stimulation [31–33]. Thus, NFAT1 partner TFs should be identified to determine the specific regulatory mechanisms of γc expression.

Another major TF activated by TCR signaling was NFκB, which appeared to play a critical role in the upregulation of γc expression. NFκB, which is a heterodimer containing p50 and p65 subunits in the cytosol, is usually suppressed by covering IκB [34, 35]. The engagement of TCR induces IκB degradation, and the active form of NFκB regulates target gene expression in the nucleus [36]. The upregulation of γc expression in T cells was substantially disturbed at the protein and mRNA levels by NFκB-specific inhibitors and in NFκB knockdown and knockout T cells. Based on the predicted NFκB-binding sites, we identified a functional NFκB-target site residing within bp -114 to -111 of the γc promoter region, which was further confirmed via a mutant promoter and ChIP assay.

**Fig. 8:**
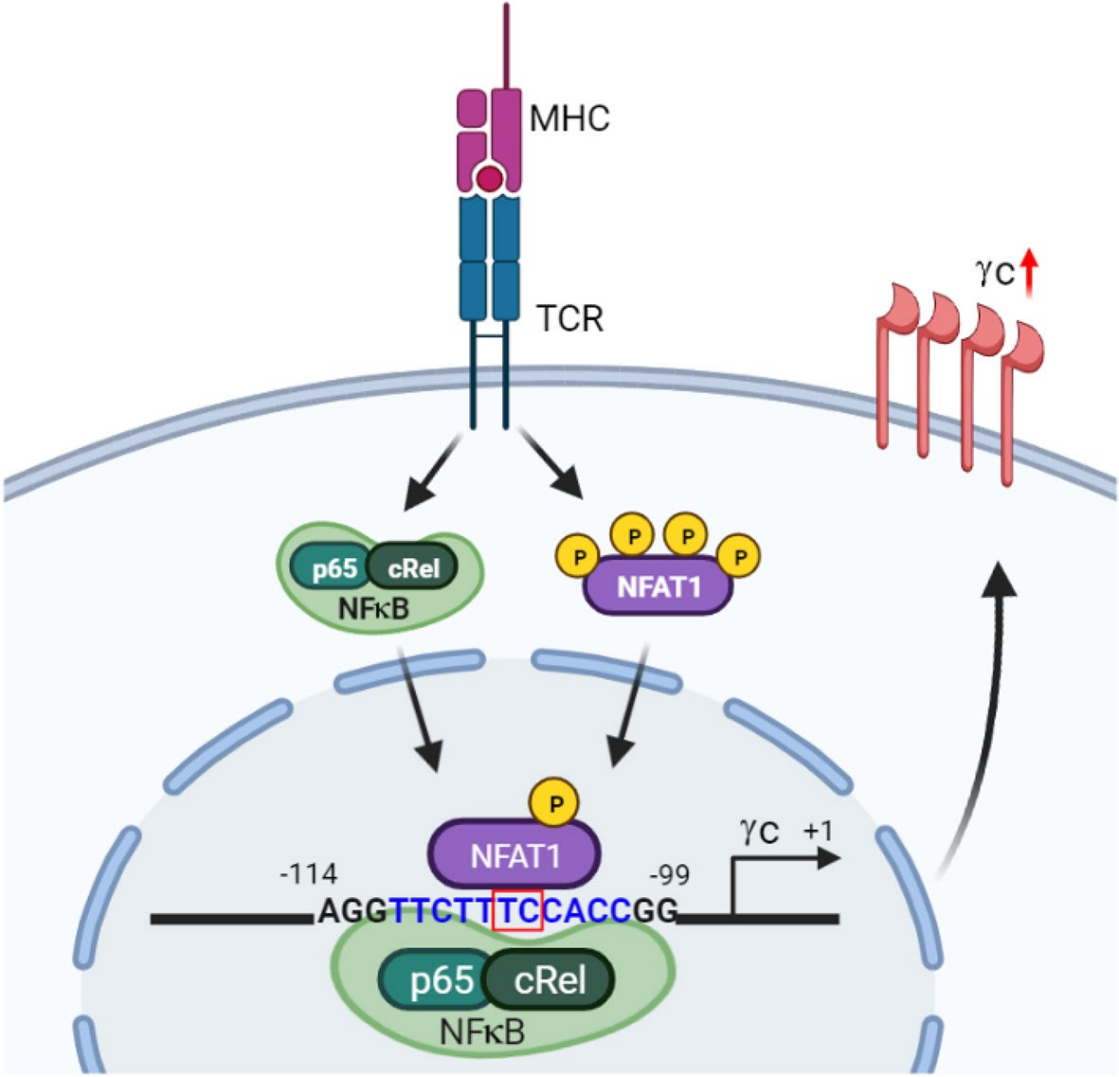
Regulation of γc expression by NFAT1 and NFκB. Summary of the regulatory mechanism of γc expression by NFAT1 and NFκB in activated T cells. The binding of TCR to peptide-MHC leads to the dephosphorylation of NFAT1 and activation of NFκB. Activation of NFκB and NFAT1 translocated to the nucleus, where it induces γc expression. In the nucleus, NFAT1 physically binds to the -111 to -101 site and NFκB binds to the -114 to -99 site on the γc promoter region and positively regulate γc gene expression

Our current results also demonstrated that both NFAT1 and NFκB are critical for the upregulation of γc expression, as the deficiency of both NFAT1 and NFκB almost ablated the upregulated γc expression. In addition, the cotransfection of NFAT1 and NFκB synergistically enhanced γc promoter activity (Fig. 8). Intriguingly, NFAT1 and NFκB appeared to occupy a similar region of the γc promoter, and mutation of the NFκB-target site within the γc promoter (GG→TT at -114 to -113) dramatically impaired the ability of not only NFκB but also NFAT1 in inducing promoter activity, and vice versa (TC→GA at -107 to -106). When considering the γc bands between the NFκB KO and NFAT1/NFκB KO samples, regulatory role of NFAT1 may exhibit redundancy in γc expression. However, synergetic effect of NFAT1/NFκB deficiency in protein and mRNA level was demonstrated by FACS analysis and RT-PCR as well as enhanced promoter activity with both TFs. These data collectively support a regulatory model of two TFs, NFAT1 and NFκB, that synergistically converge on the γc promoter to regulate transcription; however, the network and complexity with which these two TFs collaborate and interact on the γc promoter should be investigated further. Although both NFAT1 and NFκB were proved as essential TFs in regulation of γc expression, transcriptional cooperation with other factors such as NFAT2 cannot be excluded.

An important question is why γc expression was upregulated in activated T cells. Since activated T cells undergo dynamic and diverse differentiation with the reorganization of the cytoskeleton, activation of TFs, and synthesis of new proteins via several downstream pathways [37–39], it is challenging to attribute the role of increased γc expression to activated T cells. Therefore, we sought to confirm the cytokine responsiveness in T cells that overexpress only γc. While the γcTg model may not serve as a definitive demonstration of the upregulated significance of γc in activated T cells, it can be regarded as a mean to approximate the regulatory role of γc in cytokine signaling. TCR-stimulated T cells induce an optimal immune response through an immune-stimulatory positive feedback loop and regulate an excessive immune response via an immune-inhibitory negative feedback loop, thereby maintaining T cell homeostasis [38, 40–42]. Therefore, an increase in γc expression in activated T cells is expected to contribute to the feedback loop that induces an appropriate T cell response. Because the surface overexpression of γc in T cells dampened IL-7 signaling, increased γc expression most likely acted as a negative feedback system to optimize T cell responses. One possible explanation for reduction of IL-7 signaling in γcTg T cells is related to JAK3 expression. Previous reports have shown that JAK3 expression is not increased by TCR signaling [43]. Indeed, if there is an increase in γc expression without a concurrent increase in JAK3 expression, it could lead to the generation of a significant pool of JAK3-free γc molecules. These JAK3-free γc would be available to compete with JAK3-bound γc for binding to the IL-7Rα. Consequently, the formation of IL-7Rα/γc complexes involving JAK3-free γc could contribute to a decrease in the overall IL-7 signaling. This competition for binding to IL-7Rα by both JAK3-bound and JAK3-free γc molecules may underlie the reduction in IL-7 signaling. Further studies on how upregulated γc expression inhibits γc cytokine signaling are warranted.

Regulation of γc expression can affect the development and function of T, B, and NK cells [16–18, 44], and γc deficiency or mutation results in X-SCID in humans and mice, leading to immune deficiency [1, 3, 45]. The regulatory mechanisms and therapeutic uses of γc family cytokines have been studied extensively in the context of immune-related diseases [46]. However, to the best of our knowledge, studies related to the regulatory mechanism of γc expression have not been conducted, despite the fact that identifying the regulatory mechanism of γc expression assists in its application as a therapeutic strategy for immune-related diseases. Based on our results that γc expression is regulated in a NFAT1- and NFκB-dependent manner via TCR signaling and that the regulation of its expression is involved in γc cytokine signaling, we propose therapeutic applications by controlling γc expression.

## Conclusion

We demonstrated that γc expression is upregulated at the transcriptional level upon direct TCR signaling and that the activation of NFAT1 and NFκB TFs is involved in the regulation of *γc* gene expression. Identification of the regulatory mechanism of γc expression provides cues to control γc family cytokine signaling and its beneficial effects on autoimmune diseases and cancer.

### Supplementary Information

**Additional file 1.** Supplemental method.

**Additional file 2:** **Figure S1. **γc expression in T cells by TCR signaling pathway inhibition. (A) LN T cell treated with α-TCR/α-CD28 in the presence of PD980 and wortmannin specifically inhibited MEK and AKT phosphorylation by western blot analysis. (B) Upregulation of γc expression is not inhibited by inhibitors for AKT and ERK pathway. CD8^+^ T cells were cultured O/N on α-TCR/α-CD28 coated plates in presence of PD980 and Wortmannin. Surface expression level of γc and CD69 was assessed by flow cytometry. (C) Viability of LN T cells under α-TCR/α-CD28 treated with Bay11 or INCA6 dose dependent. Activated T cells were treated with NFAT1 and NFκB inhibitors (each concentration; INCA6: 0, 1, 3, 5 and 10 µM, and Bay11: 0, 1, 5 and 10 µM) in vitro. After 16 hr of incubation, cell viability was assessed using Propidium Iodide (PI) staining. (D) Western blot analysis showed that LNT cells under α-TCR/α-CD28 treated with Bay11 (3 µM) or INCA6 (5 µM) or Bay11 (2.5 µM)/INCA6 (2.5 µM) inhibited NFAT1 and p65 phosphorylation. (E) LN CD4^+^ T cells were cultured O/N on α-TCR/α-CD28 coated plates in presence of Bay11 (3 µM) and INCA6 (5 µM). Surface expression level of γc (left), CD69 (middle), and TCRβ (right) was assessed by FACS. The bar graph shows the relative surface expression γc (left), CD69 (middle), and TCRβ (right). The relative γc, CD69, and TCRβ expression were calculated as the ΔMFI associated with the inhibitor-treated group over the ΔMFI associated with the DMSO control group. Data are means ± SEM of three independent experiments. * P < 0.05, ** P < 0.01, PD; PD980, wort; wortmannin, Bay; Bay11, INCA; INCA6. **Figure S2.** NFAT1 and NFκB pathways are related to the regulation of γc expression. (A) Expression of γc protein is increased time-dependently upon PMA (12.5 ng/ml)/Iono (1 µM) stimulation. EL4 cells were stimulated with PMA/Iono for indicated time point. The γc and CD69 expression levels were determined by flow cytometry. Results are the summary of three independent experiments. (B) γc expression is specifically inhibited by treatment of 5,6-dichloro-1-beta-D-ribofuranosylbenzimidazole (DRB). Cell surface γc staining of EL4 cells incubated with DRB (50 µM) and cyclohexamide (CHX, 2 µg/ml) in presence of PMA/Iono. Surface γc and CD69 staining was assessed as mean fluorescence intensity (MFI) in EL4 cells. Data are summary of two independent experiments. (C and D) EL4 cells were cultured O/N with PMA/Ionomycin in presence of (C) MG132 (500 nM) or (D) CsA (1 ug/ml) for indicated time point. The γc expression level was measured by flow cytometry (left) and mRNA level was measured by quantitative RT-PCR analysis (right). DMSO was used as vehicle control. (E) Upregulation of γc expression is significantly inhibited by Bay11 (3 µM) or INCA6 (5 µM) and synergistically blocked by both Bay11 (2.5 µM) and INCA6 (2.5 µM). EL4 cells were stimulated with PMA/Iono in the presence or absence of the Bay11 and INCA6 as specific inhibitors for NFκB and NFAT1, respectively. The γc protein expression is analyzed by FACS and mRNA expression was determined by RT-qPCR. Fold induction of γc content was calculated to that of DMSO. DMSO was used as vehicle control. Data are means ± SEM of three independent experiments. *p < 0.05, ** p < 0.01 and *** p < 0.001. **Figure S3. **Targeted disruption of NFAT1 and NFκB gene by CRISPR/Cas9 system in EL4 cells. NFAT1, p65, cRel, NFκB KO (p65, cRel), and NFAT1/NFκB KO targeted by using CRISPR/Cas9 technology in EL4 cells. (A) Western blot analysis of γc, NFAT1, cRel expression in NFAT1 (#21 and #24) and NFκB and NFAT1/NFκB (#7 and #10) KO EL4 cells with PMA/Iono stimulation for 16hr. GAPDH was used as the loading control. Data are representative of three independent experiments. (B) The γc and CD69 expression levels in NFAT1 KO (#21 and #24), NFκB KO and NFAT1/NFκB KO (#7 and #10) EL4 cells treated with PMA/Iono for 16hr were determined by flow cytometry. (C) NFAT1, p65, and cRel mRNA levels in EL4 and EL4KO cell line by RT-qPCR analysis. Relative gene expression was calculated with *Rpl13* housekeeping gene. (D) NFAT1, p65, cRel protein levels in EL4 KO cell by western blot (left). Bar graphs show a summary of western blot data from three independent experiments. Relative gene expression was calculated with β-actin (right). Data are means ± SEM of three independent experiments. *p < 0.05, ** p < 0.01 and *** p < 0.001. **Figure S4. **NFAT1 and NFκB predicted binding regions on upstream of γc gene. ECR browser analysis of the mouse and human γc loci is shown. (A) six NFAT1 binding sites of γc upstream region motifs were predicted. (B) Eight NFκB binding sites of γc upstream region motifs were predicted. (C) HEK293 T cells were transfected with pGL4-NFAT1-luc plasmid that carry whole γc promoter region and then were stimulated with PMA/Iono for indicated time dependent. Luciferase activity in the lysates of transfected cells was determined by a dual luciferase assay. Data are means ± SEM of three independent experiments. * p < 0.05. **Figure S5. **Regulation of γc expression in mutant NFAT1/NFκB binding site in γc promoter region. The mutant EL4 cell lines (mut111; #120 and #126) were stimulated with PMA/Iono for 16 hr. (A) Western blot analysis of γc expression in mut111 (#120 and #126) and control EL4 cells. GAPDH was used as the loading control. Data are representative of three independent experiments. (B) The γc and CD69 expression level in mut111 (#120) and WT EL4 cell lines were analyzed using FACS. γc and CD69 expression (open histogram) were assessed in activated mut111 (#120) and control cell lines and overlaid with control antibody staining (shaded histogram). Bar graph is summary of three independent experiments. *** *p* < 0.001.

**Additional file 3.** NGS-seq data of mutant cell lines (mut111).

**Additional file 4.** Full immunoblot images.

## Data Availability

The datasets used and/or analyzed during the current study are available from the corresponding author upon reasonable request.

## Abbreviations

γc: Common γ chain
TCR: T cell receptor
LN: Lymph node
NFAT: Nuclear Factor of Activated T-cell
NFkB: Nuclear factor kappa-light-chain-enhancer of activated B cells
IL: Interleukin
IFN: Interferon
Tg: Transgenic
KO: Knockout
X-SCID: X-linked severe combined immunodeficiency
PMA: Phorbol 12-myristate 13-acetate
Iono: Ionomycin
DRB: 5,6-Dichloro-1-beta-D-ribofuranosylbenzimidazole
CHX: Cycloheximide
CsA: Cyclosporine A
ELISA: Enzyme-linked immunosorbent assay
ChIP: Chromatin Immunoprecipitation
Indel: Insertion and deletion
NGS: Next-generation sequencing
TFs: Transcription factors
RT-qPCR: Reverse transcription-quantitative PCR
NK: Natural killer
