# Supplementary material for: NFAT1 and NFκB regulates expression of the common γ-chain cytokine receptor in activated T cells
Source: Cell Commun Signal. 2023 Oct 30;21:309. doi: 10.1186/s12964-023-01326-7 (PMC10617197; doi:10.1186/s12964-023-01326-7)
Supplement: Supplementary file 2 — Additional file 1. Supplemental method. [file 12964_2023_1326_MOESM1_ESM.docx]

**Additional file 1: Supplementary method**

**Motif Enrichment Analysis**

Identification and sequence analysis of evolutionary conserved regions (ECRs) of the mouse and human γc was performed with the ECR Browser. A search for potential regulatory elements that have highly conserved binding sites for NFAT1 and NFκB between humans and mice in the upstream regulatory region of the γc gene was performed with the publicly available web-based tool rVISTA 2.0 and TRANSFAC database analysis.
