# Supplementary figures and images for: NFAT1 and NFκB regulates expression of the common γ-chain cytokine receptor in activated T cells
Source: Cell Commun Signal. 2023 Oct 30;21:309. doi: 10.1186/s12964-023-01326-7 (PMC10617197; doi:10.1186/s12964-023-01326-7)

## Slide 1
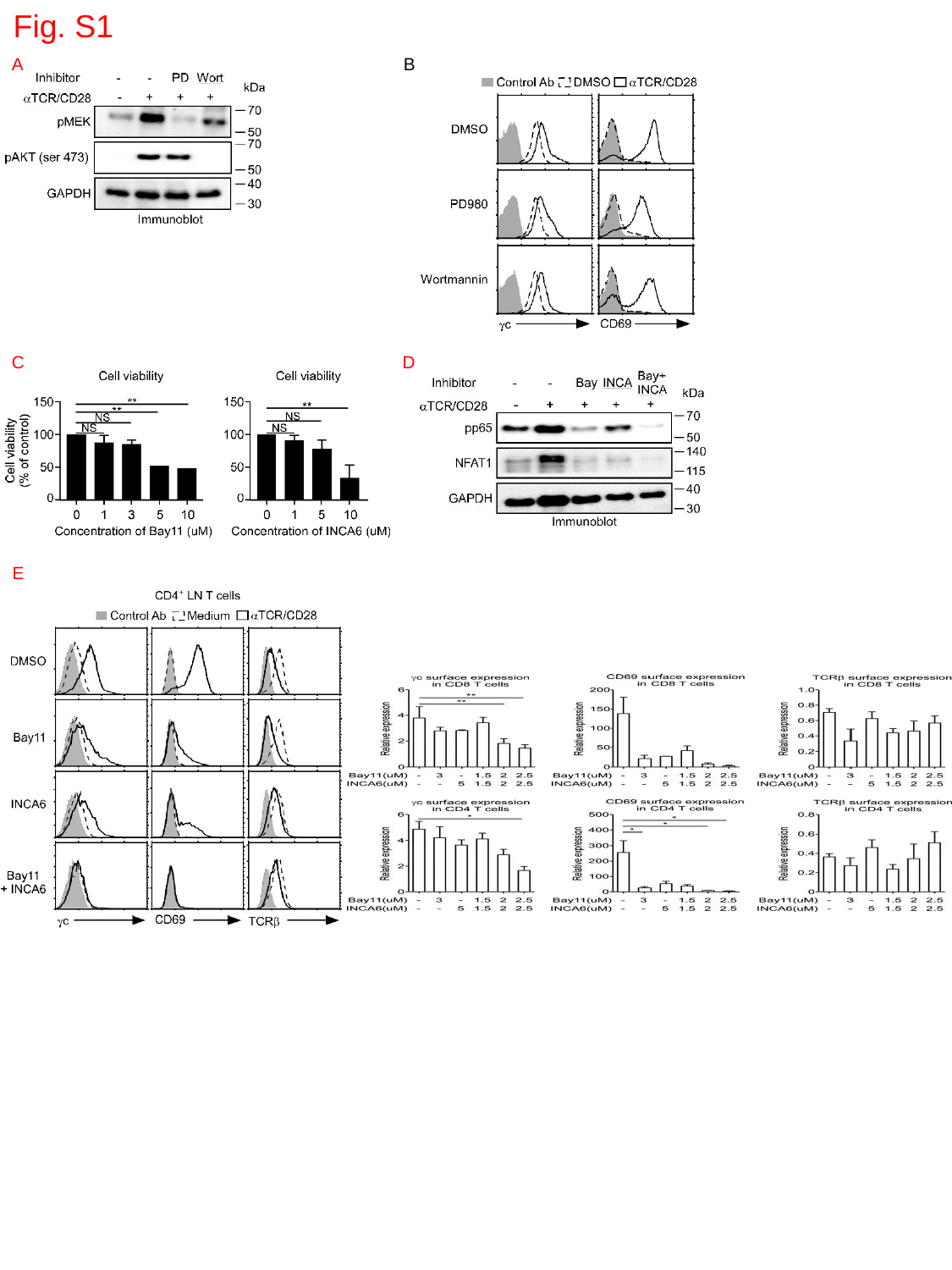

Fig. S1
A
B
C
D
E

## Slide 2
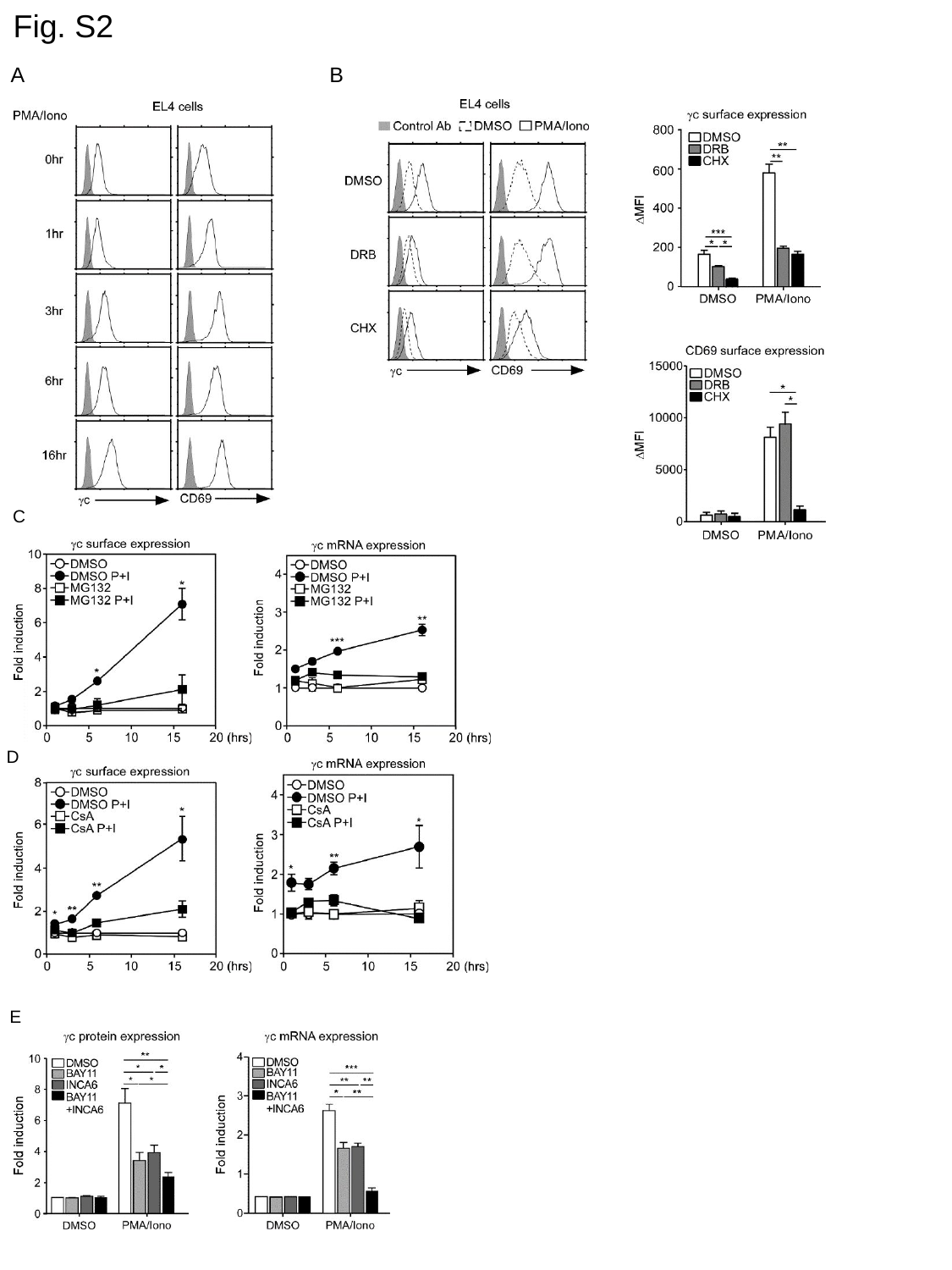

Fig. S2
A
B
C
D
E

## Slide 3
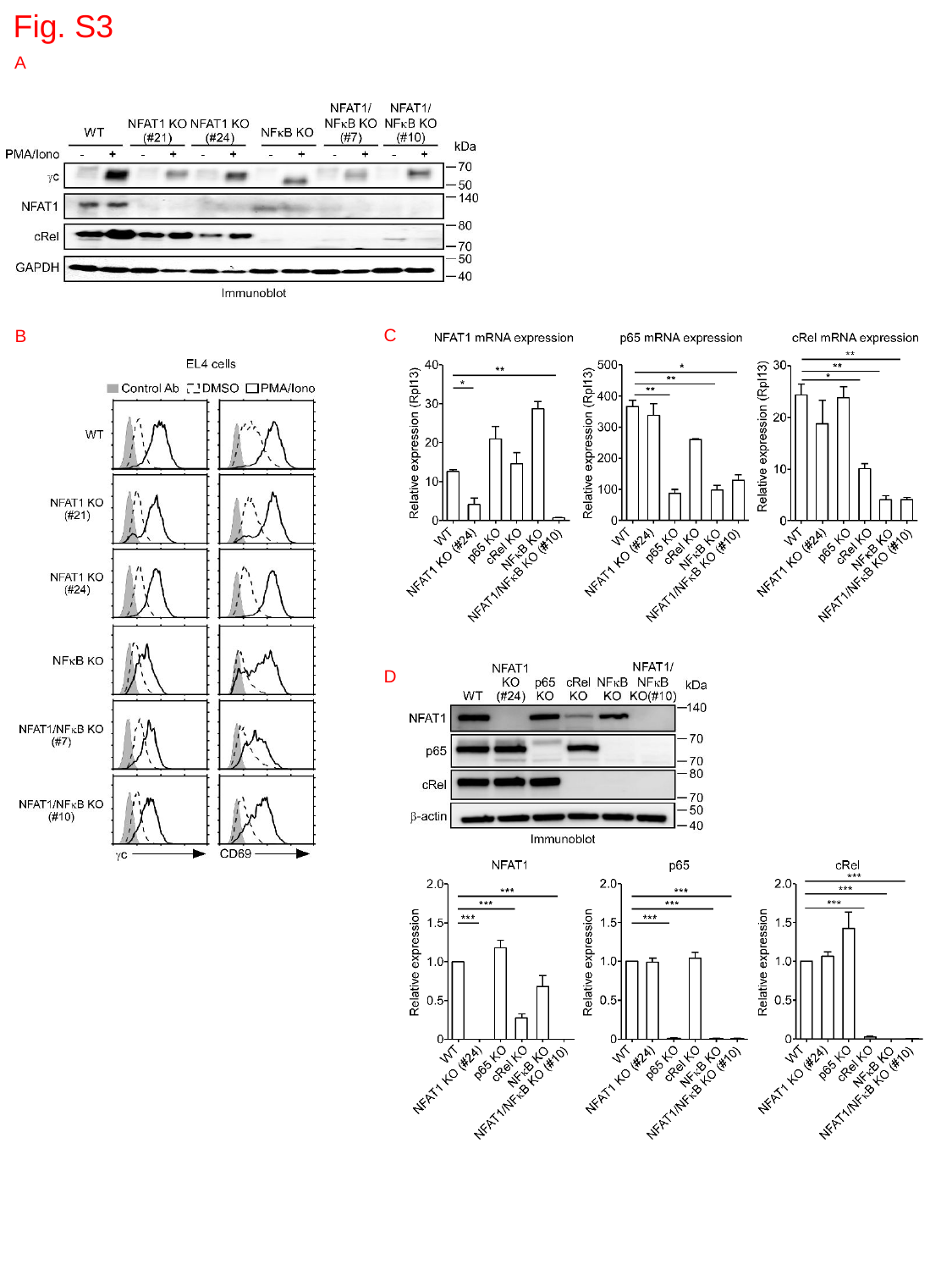

Fig. S3
A
C
B
D

## Slide 4
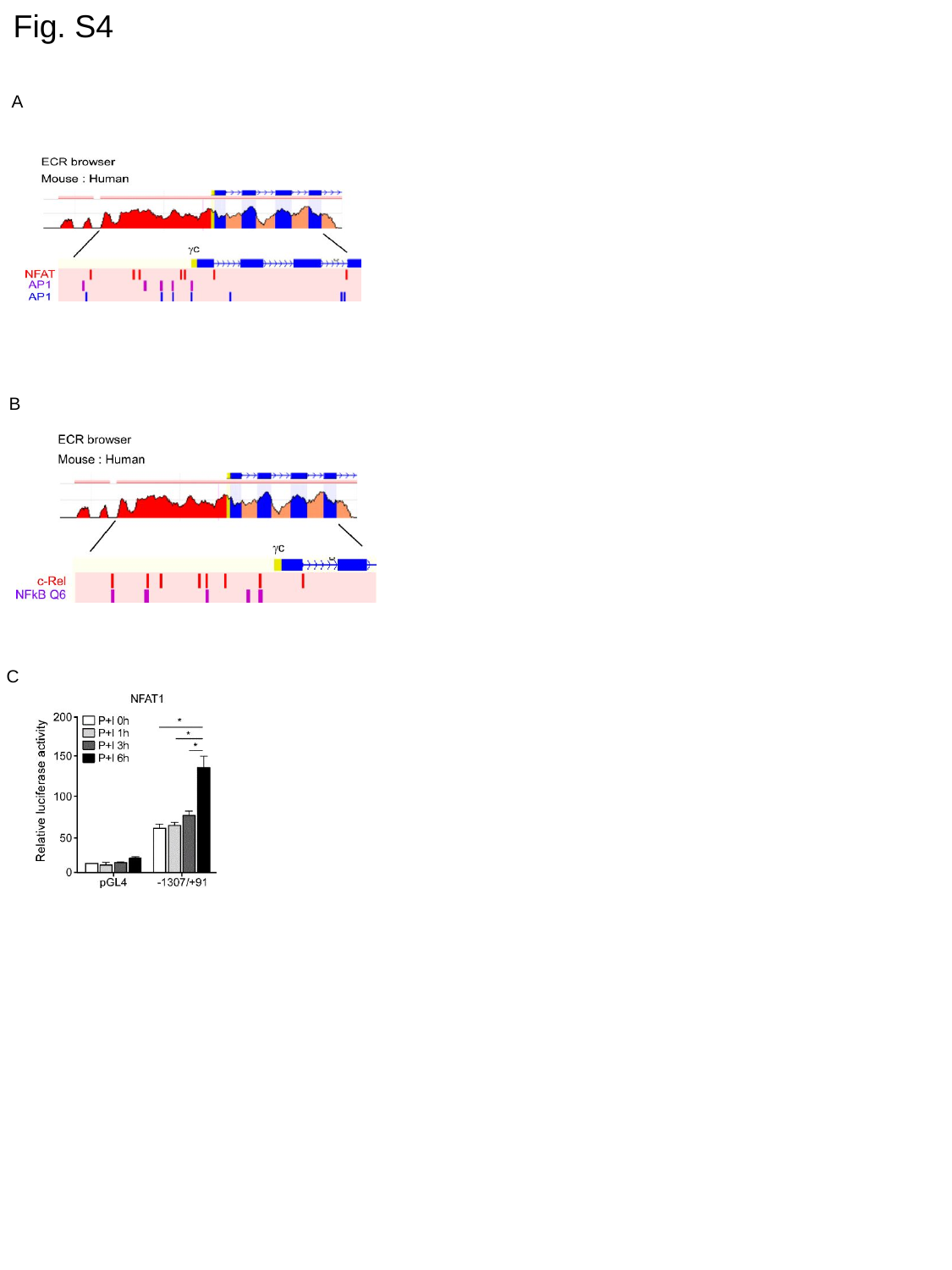

Fig. S4
A
B
C

## Slide 5
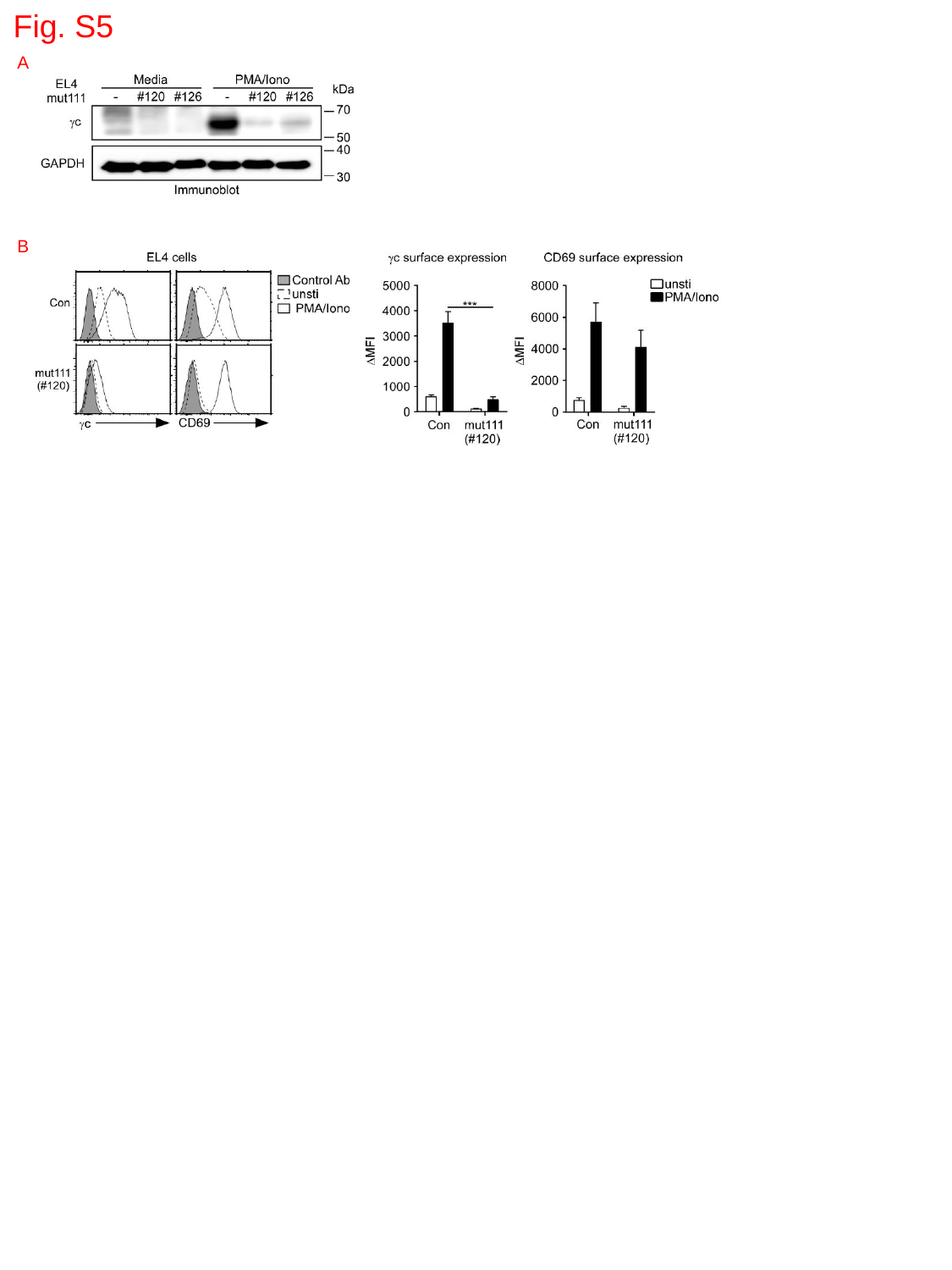

Fig. S5
A
B

Supplement: Supplementary file 3 — Additional file 2: Figure S1. γc expression in T cells by TCR signaling pathway inhibition. (A) LN T cell treated with α-TCR/α-CD28 in the presence of PD980 and wortmannin specifically inhibited MEK and AKT phosphorylation by western blot analysis. (B) Upregulation of γc expression is not inhibited by inhibitors for AKT and ERK pathway. CD8+ T cells were cultured O/N on α-TCR/α-CD28 coated plates in presence of PD980 and Wortmannin. Surface expression level of γc and CD69 was assessed by flow cytometry. (C) Viability of LN T cells under α-TCR/α-CD28 treated with Bay11 or INCA6 dose dependent. Activated T cells were treated with NFAT1 and NFκB inhibitors (each concentration; INCA6: 0, 1, 3, 5 and 10 µM, and Bay11: 0, 1, 5 and 10 µM) in vitro. After 16 hr of incubation, cell viability was assessed using Propidium Iodide (PI) staining. (D) Western blot analysis showed that LNT cells under α-TCR/α-CD28 treated with Bay11 (3 µM) or INCA6 (5 µM) or Bay11 (2.5 µM)/INCA6 (2.5 µM) inhibited NFAT1 and p65 phosphorylation. (E) LN CD4+ T cells were cultured O/N on α-TCR/α-CD28 coated plates in presence of Bay11 (3 µM) and INCA6 (5 µM). Surface expression level of γc (left), CD69 (middle), and TCRβ (right) was assessed by FACS. The bar graph shows the relative surface expression γc (left), CD69 (middle), and TCRβ (right). The relative γc, CD69, and TCRβ expression were calculated as the ΔMFI associated with the inhibitor-treated group over the ΔMFI associated with the DMSO control group. Data are means ± SEM of three independent experiments. * P < 0.05, ** P < 0.01, PD; PD980, wort; wortmannin, Bay; Bay11, INCA; INCA6. Figure S2. NFAT1 and NFκB pathways are related to the regulation of γc expression. (A) Expression of γc protein is increased time-dependently upon PMA (12.5 ng/ml)/Iono (1 µM) stimulation. EL4 cells were stimulated with PMA/Iono for indicated time point. The γc and CD69 expression levels were determined by flow cytometry. Results are the summary of three independe [file 12964_2023_1326_MOESM2_ESM.pptx]
