# Supplementary material for: NFAT1 and NFκB regulates expression of the common γ-chain cytokine receptor in activated T cells
Source: Cell Commun Signal. 2023 Oct 30;21:309. doi: 10.1186/s12964-023-01326-7 (PMC10617197; doi:10.1186/s12964-023-01326-7)
Supplement: Supplementary file 4 — Additional file 3. NGS-seq data of mutant cell lines (mut111). [file 12964_2023_1326_MOESM3_ESM.pdf]

NGS seq_Result

EL4 111mut cell line

Clone (#120, 126)

#120 Cas-Analyzer

Cas-Analyzer

A JavaScript-based instant assessment tool for high-throughput sequencing data for genome edited cells.

Thanks to the improvements in the newest JavaScript engines in the most recent web browsers, the JavaScript based internal algorithm of Cas-Analyzer completely runs on the client-side so that large amounts of sequencing data do not need to be uploaded to the server. Currently, Cas-Analyzer supports various single nucleases (SpCas9, StCas9, NmCas9, SaCas9, CjCas9, and AsCpf1/LbCpf1) and paired nucleases (ZFNs, TALENs, Cas9 nickases, and dCas9-FokI nucleases).

Citation info: Park J. *et al*. Cas-Analyzer: an online tool for assessing genome editing results using NGS data. *Bioinformatics* **33**, 286-288 (2017).

For the ones who would like to clarify the errors derived from DNA polymerase during PCR or sequencing process, comparison of treated sample and negative control (e.g. untreated sample) is recommended.

Please input your data in below form, or **download an example data here**.

Sequencing Data

File Type:

Single-end read or fastq-joined file

Single Read File (fastq or gzipped fastq):

파일 선택 29.fastqjoin

Basic Information

Full reference sequence (5' to 3'):

Caccttagagcagaacccaaatctccctggggacttagcttatgtcactgaacacatttaccaaccccccctctctctacagcgtggtttctaaggttctttccaccggaagctacgacaaaaggaaatgtatgggtggggagggcttgtgggagagtggttcagggttctgacacagactacacccagagaaagaagagcaagcaccatgttgaaa

Nuclease Type:

Single nuclease

Select Nuclease:

SpCas9 from Streptococcus pyogenes: 5'-NGG-3'

Target DNA sequence (5' to 3', without PAM sequence):

Gtttctaaggttctttccac

(Optional) Donor DNA sequence for homology directed repair (HDR) (5' to 3')

ccctctctctacagcgtggtttctaaggttcttgacaccggaagctacgacaaaaggaaatgtatgggtggggagggcttgtgggagagtggttcagggt

Analysis Parameters

Comparison range (R) [?]

70

or use both ends

Minimum frequency (n) [?]

1

(Optional) WT marker (r) [?]

5

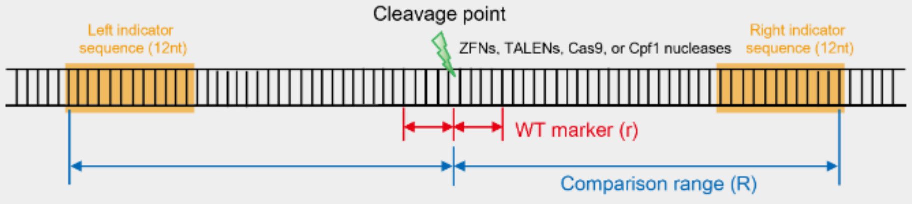

Submit

#120 HDR frequency

CRISPR RGEN Tools | About | Cas-OFFinder | Microhomology | Cas-Designer | Database | Analyzer | Digenome-Seq | Base Editing | Prime Editing

Please input your data in below form, or download an example data here.

Input Summary

File name		
29.fastqjoin		
**WT sequence**<br>**(blue: indicator sequences at each ends of comparision range, green: crRNA sequence, red: WT marker sequence)**		
CACCTTAGAGCAGAACCCAAATCTCCCTGGGGA**CTTAGCTTATGTCAC**TGAACACATTTACCAACCCCCCCTCTCTCTACAGCGTG**GTTTCTAAGGTT**CTTTCCACCGGAAGCTACGACAAAAGGAAATGTATGGGTGGGGAGGGCTTGTGGGAGAGT**GGTTCAGGGTTCTGA**CACAGACTACACCCAGAGAAAGAAGAGCAAGCACCATGTTGAAA		
**crRNA sequence**		
GTTTCTAAGGTTCTTTCCAC		
**Comparison range (R) [?]**	**Minimum frequency (n) [?]**	**WT marker (r) [?]**
70	1	5

Run again with different values

Result Summary

Total Sequences	With both indicator sequences	More than minimum frequency	Insertions	Deletions	Indel frequency	HDR frequency
2717	2276	2062	3	18	21 (1.0%)	2036 (98.7%)

Insertions and Deletions

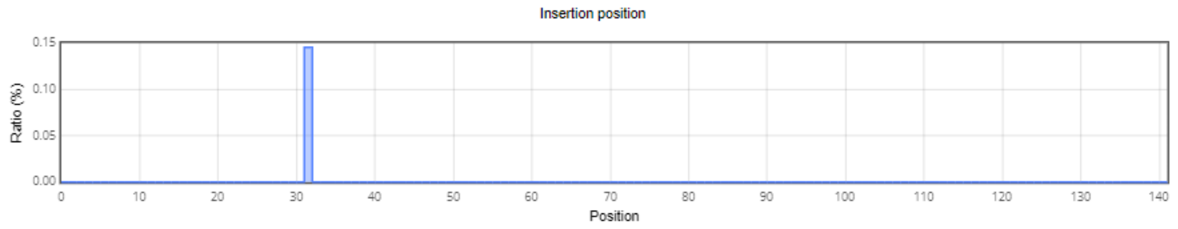

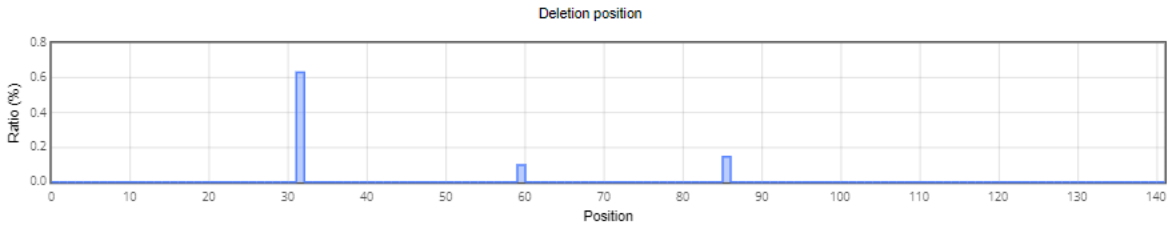

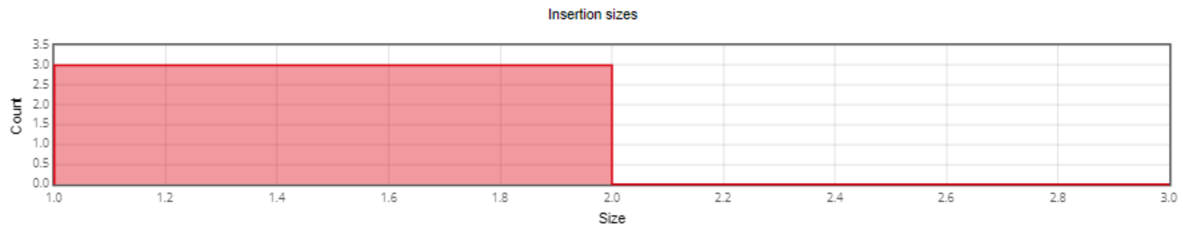

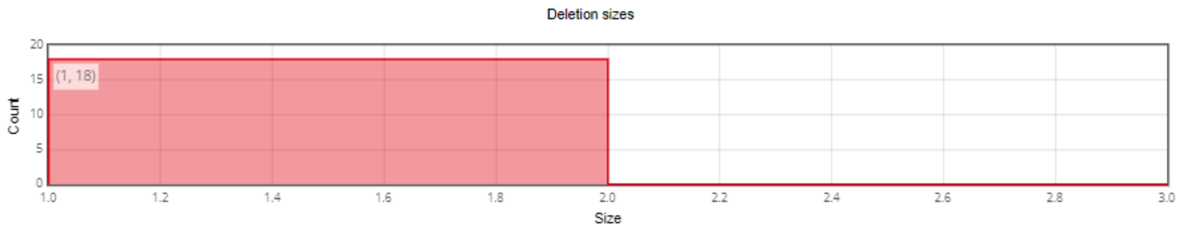

#120 Sequence information

Sequence Information

All | WT and Substitutions | Insertions | Deletions | ☐ Show HDR only | Download table

<table>
<tr><th>ID</th><th>Sequence</th><th>Length</th><th>Count</th><th>Type</th><th>HDR</th></tr>
<tr><td>1</td><td>CTTAGCTTATGTCACTGAACACATTTACCAACCCCCCCTCTCTCTACAGCGTGGTTTCTAAGGTTCTTTCCACCGGAAGCTACGACAAAAGGAAATGTATGGGTGGGGAGGGCTTGTGGGAGAGTGGTTCAGGGTTCTGA
||||||||||||||||||||||||||||||||||||||||||||||||||||||||||||||||||||..||||||||||||||||||||||||||||||||||||||||||||||||||||||||||||||||||||||
CTTAGCTTATGTCACTGAACACATTTACCAACCCCCCCTCTCTCTACAGCGTGGTTTCTAAGGTTCTTGACACCGGAAGCTACGACAAAAGGAAATGTATGGGTGGGGAGGGCTTGTGGGAGAGTGGTTCAGGGTTCTGA</td><td>140</td><td>1769</td><td>WT or Sub</td><td>O</td></tr>
<tr><td>2</td><td>CTTAGCTTATGTCACTGAACACATTTACCAACCCCCCCTCTCTCTACAGCGTGGTTTCTAAGGTTCTTTCCACCGGAAGCTACGACAAAAGGAAATGTATGGGTGGGGAGGGCTTGTGGGAGAGTGGTTCAGGGTTCTGA
||||||||||||||||||||||||||||||| ||||||||||||||||||||||||||||||||||||..||||||||||||||||||||||||||||||||||||||||||||||||||||||||||||||||||||||
CTTAGCTTATGTCACTGAACACATTTACCAA-CCCCCCTCTCTCTACAGCGTGGTTTCTAAGGTTCTTGACACCGGAAGCTACGACAAAAGGAAATGTATGGGTGGGGAGGGCTTGTGGGAGAGTGGTTCAGGGTTCTGA</td><td>139</td><td>9</td><td>Del</td><td>O</td></tr>
<tr><td>3</td><td>CTTAGCTTATGTCACTGAACACATTTACCAACCCCCCCTCTCTCTACAGCGTGGTTTCTAAGGTTCTTTCCACCGGAAGCTACGACAAAAGGAAATGTATGGGTGGGGAGGGCTTGTGGGAGAGTGGTTCAGGGTTCTGA
|||||||||||||.||||||||||||||||||||||||||||||||||||||||||||||||||||||..||||||||||||||||||||||||||||||||||||||||||||||||||||||||||||||||||||||
CTTAGCTTATGTCGCTGAACACATTTACCAACCCCCCCTCTCTCTACAGCGTGGTTTCTAAGGTTCTTGACACCGGAAGCTACGACAAAAGGAAATGTATGGGTGGGGAGGGCTTGTGGGAGAGTGGTTCAGGGTTCTGA</td><td>140</td><td>7</td><td>WT or Sub</td><td>O</td></tr>
<tr><td>4</td><td>CTTAGCTTATGTCACTGAACACATTTACCAACCCCCCCTCTCTCTACAGCGTGGTTTCTAAGGTTCTTTCCACCGGAAGCTACGACAAAAGGAAATGTATGGGTGGGGAGGGCTTGTGGGAGAGTGGTTCAGGGTTCTGA
|||||||||||.||||||||||||||||||||||||||||||||||||||||||||||||||||||||..||||||||||||||||||||||||||||||||||||||||||||||||||||||||||||||||||||||
CTTAGCTTATGGCACTGAACACATTTACCAACCCCCCCTCTCTCTACAGCGTGGTTTCTAAGGTTCTTGACACCGGAAGCTACGACAAAAGGAAATGTATGGGTGGGGAGGGCTTGTGGGAGAGTGGTTCAGGGTTCTGA</td><td>140</td><td>7</td><td>WT or Sub</td><td>O</td></tr>
<tr><td>5</td><td>CTTAGCTTATGTCACTGAACACATTTACCAACCCCCCCTCTCTCTACAGCGTGGTTTCTAAGGTTCTTTCCACCGGAAGCTACGACAAAAGGAAATGTATGGGTGGGGAGGGCTTGTGGGAGAGTGGTTCAGGGTTCTGA
||||||||||||||||||||||||||||.|||||||||||||||||||||||||||||||||||||||..||||||||||||||||||||||||||||||||||||||||||||||||||||||||||||||||||||||
CTTAGCTTATGTCACTGAACACATTTACAAACCCCCCCTCTCTCTACAGCGTGGTTTCTAAGGTTCTTGACACCGGAAGCTACGACAAAAGGAAATGTATGGGTGGGGAGGGCTTGTGGGAGAGTGGTTCAGGGTTCTGA</td><td>140</td><td>6</td><td>WT or Sub</td><td>O</td></tr>
<tr><td>6</td><td>CTTAGCTTATGTCACTGAACACATTTACCAACCCCCCCTCTCTCTACAGCGTGGTTTCTAAGGTTCTTTCCACCGGAAGCTACGACAAAAGGAAATGTATGGGTGGGGAGGGCTTGTGGGAGAGTGGTTCAGGGTTCTGA
|||||||||||||||||||||||||||||||||||||.||||||||||||||||||||||||||||||..||||||||||||||||||||||||||||||||||||||||||||||||||||||||||||||||||||||
CTTAGCTTATGTCACTGAACACATTTACCAACCCCCCTTCTCTCTACAGCGTGGTTTCTAAGGTTCTTGACACCGGAAGCTACGACAAAAGGAAATGTATGGGTGGGGAGGGCTTGTGGGAGAGTGGTTCAGGGTTCTGA</td><td>140</td><td>6</td><td>WT or Sub</td><td>O</td></tr>
<tr><td>7</td><td>CTTAGCTTATGTCACTGAACACATTTACCAACCCCCCCTCTCTCTACAGCGTGGTTTCTAAGGTTCTTTCCACCGGAAGCTACGACAAAAGGAAATGTATGGGTGGGGAGGGCTTGTGGGAGAGTGGTTCAGGGTTCTGA
||||||||||||||||||||||||||||||||||||||||||||||||||||||||||||||||||||..|||||||||||||||||||||||||||||||||.||||||||||||||||||||||||||||||||||||
CTTAGCTTATGTCACTGAACACATTTACCAACCCCCCCTCTCTCTACAGCGTGGTTTCTAAGGTTCTTGACACCGGAAGCTACGACAAAAGGAAATGTATGGGGGGGGAGGGCTTGTGGGAGAGTGGTTCAGGGTTCTGA</td><td>140</td><td>6</td><td>WT or Sub</td><td>O</td></tr>
<tr><td>8</td><td>CTTAGCTTATGTCACTGAACACATTTACCAACCCCCCCTCTCTCTACAGCGTGGTTTCTAAGGTTCTTTCCACCGGAAGCTACGACAAAAGGAAATGTATGGGTGGGGAGGGCTTGTGGGAGAGTGGTTCAGGGTTCTGA
||||||||.|||||||||||||||||||||||||||||||||||||||||||||||||||||||||||..||||||||||||||||||||||||||||||||||||||||||||||||||||||||||||||||||||||
CTTAGCTTGTGTCACTGAACACATTTACCAACCCCCCCTCTCTCTACAGCGTGGTTTCTAAGGTTCTTGACACCGGAAGCTACGACAAAAGGAAATGTATGGGTGGGGAGGGCTTGTGGGAGAGTGGTTCAGGGTTCTGA</td><td>140</td><td>6</td><td>WT or Sub</td><td>O</td></tr>
<tr><td>9</td><td>CTTAGCTTATGTCACTGAACACATTTACCAACCCCCCCTCTCTCTACAGCGTGGTTTCTAAGGTTCTTTCCACCGGAAGCTACGACAAAAGGAAATGTATGGGTGGGGAGGGCTTGTGGGAGAGTGGTTCAGGGTTCTGA
||||||||||||||.|||||||||||||||||||||||||||||||||||||||||||||||||||||..||||||||||||||||||||||||||||||||||||||||||||||||||||||||||||||||||||||
CTTAGCTTATGTCAGTGAACACATTTACCAACCCCCCCTCTCTCTACAGCGTGGTTTCTAAGGTTCTTGACACCGGAAGCTACGACAAAAGGAAATGTATGGGTGGGGAGGGCTTGTGGGAGAGTGGTTCAGGGTTCTGA</td><td>140</td><td>6</td><td>WT or Sub</td><td>O</td></tr>
<tr><td>10</td><td>CTTAGCTTATGTCACTGAACACATTTACCAACCCCCCCTCTCTCTACAGCGTGGTTTCTAAGGTTCTTTCCACCGGAAGCTACGACAAAAGGAAATGTATGGGTGGGGAGGGCTTGTGGGAGAGTGGTTCAGGGTTCTGA
||||||||||||||||||||||||||||||||||||||||||||||||||||||||||||||||||||..|||||||||||||||||||||||||||||||||||||||||||||.||||||||||||||||||||||||
CTTAGCTTATGTCACTGAACACATTTACCAACCCCCCCTCTCTCTACAGCGTGGTTTCTAAGGTTCTTGACACCGGAAGCTACGACAAAAGGAAATGTATGGGTGGGGAGGGCTTATGGGAGAGTGGTTCAGGGTTCTGA</td><td>140</td><td>5</td><td>WT or Sub</td><td>O</td></tr>
</table>

#126 Cas-Analyzer

CRISPR RGEN Tools | About | Cas-OFFinder | Microhomology | Cas-Designer | Database | Analyzer | Digenome-Seq | Base Editing | Prime Editing

Please input your data in below form, or **download an example data here**.

Sequencing Data

File Type:

Single-end read or fastq-joined file

Single Read File (fastq or gzipped fastq):

파일 선택 34.fastqjoin

Basic Information

Full reference sequence (5' to 3'):

Caccttagagcagaacccaaatctccctggggacttagcttatgtcactgaacacatttaccaaccccccctctctctacagcgtggtttctaaggttctttccaccggaagctacgacaaaaggaaatg
tatgggtggggagggcttgtgggagagtggttcagggttctgacacagactacacccagagaaagaagagcaagcaccatgttgaaa

Nuclease Type:

Single nuclease

Select Nuclease:

SpCas9 from Streptococcus pyogenes: 5'-NGG-3'

Target DNA sequence (5' to 3', without PAM sequence):

Gtttctaaggttctttccac

(Optional) Donor DNA sequence for homology directed repair (HDR) (5' to 3')

ccctctctctacagcgtggtttctaaggttcttgacaccggaagctacgacaaaaggaaatgtatgggtggggagggcttgtgggagagtggttcagggt

Analysis Parameters

Comparison range (R) [?]

70

or use both ends

Minimum frequency (n) [?]

1

(Optional) WT marker (r) [?]

5

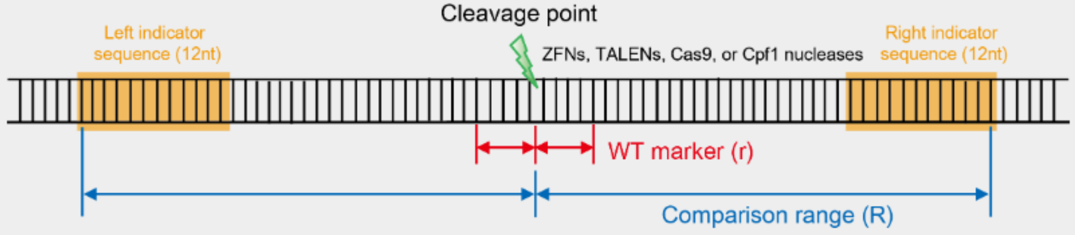

Submit

#126 HDR frequency

CRISPR RGEN Tools | About | Cas-OFFinder | Microhomology | Cas-Designer | Database | Analyzer | Digenome-Seq | Base Editing | Prime Editing

Please input your data in below form, or download an example data here.

Input Summary

File name
34.fastqjoin
**WT sequence** (blue: indicator sequences at each ends of comparision range, green: crRNA sequence, red: WT marker sequence)
CACCTTAGAGCAGAACCCAAATCTCCCTGGGGA**CTTAGCTTATGTCAC**TGAACACATTTACCAACCCCCCCTCTCTCTACAGCGTG**GTTTCTAAGGTT****CTTTCCACCG**GAAGCTACGACAAAAGGAAATGTATGGGTGGGGAGGGCTTGTGGGAGAGT**GGTTCAGGGTTCTGA**CACAGACTACACCCAGAGAAAGAAGAGCAAGCACCATGTTGAAA
**crRNA sequence**
GTTTCTAAGGTTCTTTCCAC

Comparison range (R) [?]	Minimum frequency (n) [?]	WT marker (r) [?]
70	1	5

Run again with different values

Result Summary

Total Sequences	With both indicator sequences	More than minimum frequency	Insertions	Deletions	Indel frequency	HDR frequency
4280	2769	2544	3	27	30 (1.2%)	2410 (94.7%)

Insertions and Deletions

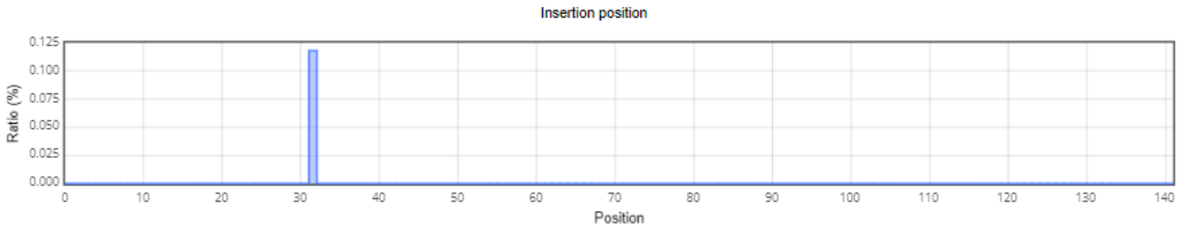

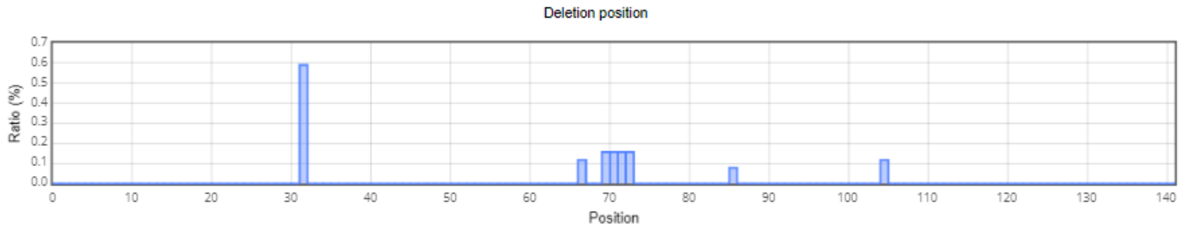

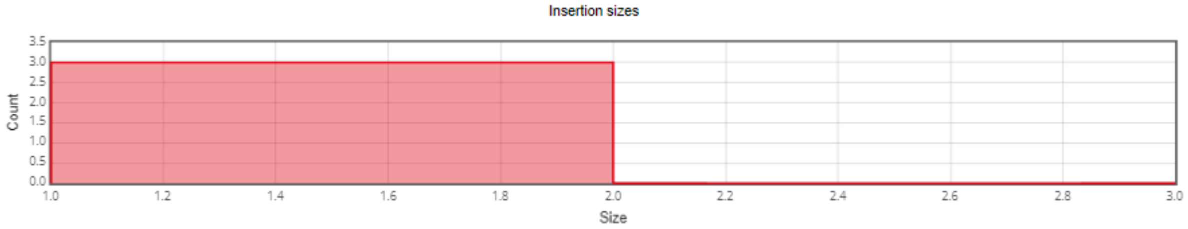

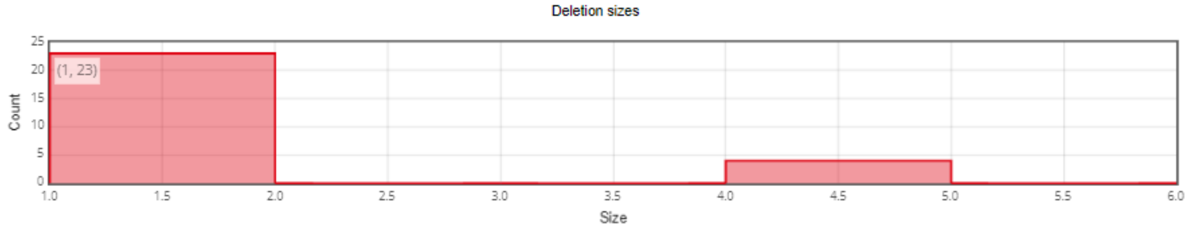

#126 Sequence information

CRISPR
RGEN Tools

About Cas-OFFinder Microhomology Cas-Designer Database Analyzer Digenome-Seq Base Editing Prime Editing

Sequence Information

All | WT and Substitutions | Insertions | Deletions | Show HDR only | Download table

<table>
<tr><th>ID</th><th>Sequence</th><th>Length</th><th>Count</th><th>Type</th><th>HDR</th></tr>
<tr><td>1</td><td>CTTAGCTTATGTCACTGAACACATTTACCAACCCCCCCTCTCTCTACAGCGTGGTTTCTAAGGTTCTTTCCACCGGAAGCTACGACAAAAGGAAATGTATGGGTGGGGAGGGCTTGTGGGAGAGTGGTTCAGGGTTCTGA
||||||||||||||||||||||||||||||||||||||||||||||||||||||||||||||||||||..||||||||||||||||||||||||||||||||||||||||||||||||||||||||||||||||||||||
CTTAGCTTATGTCACTGAACACATTTACCAACCCCCCCTCTCTCTACAGCGTGGTTTCTAAGGTTCTTGACACCGGAAGCTACGACAAAAGGAAATGTATGGGTGGGGAGGGCTTGTGGGAGAGTGGTTCAGGGTTCTGA</td><td>140</td><td>2074</td><td>WT or Sub</td><td>O</td></tr>
<tr><td>2</td><td>CTTAGCTTATGTCACTGAACACATTTACCAACCCCCCCTCTCTCTACAGCGTGGTTTCTAAGGTTCTTTCCACCGGAAGCTACGACAAAAGGAAATGTATGGGTGGGGAGGGCTTGTGGGAGAGTGGTTCAGGGTTCTGA
||||||||||||||||||||||||||||||||||||||||||||||||||||||||||||||||||||||||||||||||||||||||||||||||||||||||||||||||||||||||||||||||||||||||||||
CTTAGCTTATGTCACTGAACACATTTACCAACCCCCCCTCTCTCTACAGCGTGGTTTCTAAGGTTCTTTCCACCGGAAGCTACGACAAAAGGAAATGTATGGGTGGGGAGGGCTTGTGGGAGAGTGGTTCAGGGTTCTGA</td><td>140</td><td>106</td><td>WT or Sub</td><td>X</td></tr>
<tr><td>3</td><td>CTTAGCTTATGTCACTGAACACATTTACCAACCCCCCCTCTCTCTACAGCGTGGTTTCTAAGGTTCTTTCCACCGGAAGCTACGACAAAAGGAAATGTATGGGTGGGGAGGGCTTGTGGGAGAGTGGTTCAGGGTTCTGA
|||||||||||||||||||||||||||||||||||||.||||||||||||||||||||||||||||||..||||||||||||||||||||||||||||||||||||||||||||||||||||||||||||||||||||||
CTTAGCTTATGTCACTGAACACATTTACCAACCCCCCTTCTCTCTACAGCGTGGTTTCTAAGGTTCTTGACACCGGAAGCTACGACAAAAGGAAATGTATGGGTGGGGAGGGCTTGTGGGAGAGTGGTTCAGGGTTCTGA</td><td>140</td><td>10</td><td>WT or Sub</td><td>O</td></tr>
<tr><td>4</td><td>CTTAGCTTATGTCACTGAACACATTTACCAACCCCCCCTCTCTCTACAGCGTGGTTTCTAAGGTTCTTTCCACCGGAAGCTACGACAAAAGGAAATGTATGGGTGGGGAGGGCTTGTGGGAGAGTGGTTCAGGGTTCTGA
||||||||.|||||||||||||||||||||||||||||||||||||||||||||||||||||||||||..||||||||||||||||||||||||||||||||||||||||||||||||||||||||||||||||||||||
CTTAGCTTGTGTCACTGAACACATTTACCAACCCCCCCTCTCTCTACAGCGTGGTTTCTAAGGTTCTTGACACCGGAAGCTACGACAAAAGGAAATGTATGGGTGGGGAGGGCTTGTGGGAGAGTGGTTCAGGGTTCTGA</td><td>140</td><td>10</td><td>WT or Sub</td><td>O</td></tr>
<tr><td>5</td><td>CTTAGCTTATGTCACTGAACACATTTACCAACCCCCCCTCTCTCTACAGCGTGGTTTCTAAGGTTCTTTCCACCGGAAGCTACGACAAAAGGAAATGTATGGGTGGGGAGGGCTTGTGGGAGAGTGGTTCAGGGTTCTGA
||||||||||||||||||||||||||||||| ||||||||||||||||||||||||||||||||||||..||||||||||||||||||||||||||||||||||||||||||||||||||||||||||||||||||||||
CTTAGCTTATGTCACTGAACACATTTACCAA-CCCCCCTCTCTCTACAGCGTGGTTTCTAAGGTTCTTGACACCGGAAGCTACGACAAAAGGAAATGTATGGGTGGGGAGGGCTTGTGGGAGAGTGGTTCAGGGTTCTGA</td><td>139</td><td>9</td><td>Del</td><td>O</td></tr>
<tr><td>6</td><td>CTTAGCTTATGTCACTGAACACATTTACCAACCCCCCCTCTCTCTACAGCGTGGTTTCTAAGGTTCTTTCCACCGGAAGCTACGACAAAAGGAAATGTATGGGTGGGGAGGGCTTGTGGGAGAGTGGTTCAGGGTTCTGA
|||||||||||.||||||||||||||||||||||||||||||||||||||||||||||||||||||||..||||||||||||||||||||||||||||||||||||||||||||||||||||||||||||||||||||||
CTTAGCTTATGGCACTGAACACATTTACCAACCCCCCCTCTCTCTACAGCGTGGTTTCTAAGGTTCTTGACACCGGAAGCTACGACAAAAGGAAATGTATGGGTGGGGAGGGCTTGTGGGAGAGTGGTTCAGGGTTCTGA</td><td>140</td><td>8</td><td>WT or Sub</td><td>O</td></tr>
<tr><td>7</td><td>CTTAGCTTATGTCACTGAACACATTTACCAACCCCCCCTCTCTCTACAGCGTGGTTTCTAAGGTTCTTTCCACCGGAAGCTACGACAAAAGGAAATGTATGGGTGGGGAGGGCTTGTGGGAGAGTGGTTCAGGGTTCTGA
||||||||||||||||||||||||||||||||||||||||||||||||||||||||||||||||||||..|||||||||||||||||||||||||||||||||.||||||||||||||||||||||||||||||||
CTTAGCTTATGTCACTGAACACATTTACCAACCCCCCCTCTCTCTACAGCGTGGTTTCTAAGGTTCTTGACACCGGAAGCTACGACAAAAGGAAATGTATGGGGGGGGAGGGCTTGTGGGAGAGTGGTTCAGGGTTCTGA</td><td>140</td><td>7</td><td>WT or Sub</td><td>O</td></tr>
<tr><td>8</td><td>CTTAGCTTATGTCACTGAACACATTTACCAACCCCCCCTCTCTCTACAGCGTGGTTTCTAAGGTTCTTTCCACCGGAAGCTACGACAAAAGGAAATGTATGGGTGGGGAGGGCTTGTGGGAGAGTGGTTCAGGGTTCTGA
||||||||||||||.|||||||||||||||||||||||||||||||||||||||||||||||||||||..||||||||||||||||||||||||||||||||||||||||||||||||||||||||||||||||||||||
CTTAGCTTATGTCAGTGAACACATTTACCAACCCCCCCTCTCTCTACAGCGTGGTTTCTAAGGTTCTTGACACCGGAAGCTACGACAAAAGGAAATGTATGGGTGGGGAGGGCTTGTGGGAGAGTGGTTCAGGGTTCTGA</td><td>140</td><td>7</td><td>WT or Sub</td><td>O</td></tr>
<tr><td>9</td><td>CTTAGCTTATGTCACTGAACACATTTACCAACCCCCCCTCTCTCTACAGCGTGGTTTCTAAGGTTCTTTCCACCGGAAGCTACGACAAAAGGAAATGTATGGGTGGGGAGGGCTTGTGGGAGAGTGGTTCAGGGTTCTGA
||||||||||||||||||||||||||||||||||||||||||||||||||||||||||||||||||||..|||||||||||||||||||||||||||||||||||||||||||||||||||||||||||||||.||||||
CTTAGCTTATGTCACTGAACACATTTACCAACCCCCCCTCTCTCTACAGCGTGGTTTCTAAGGTTCTTGACACCGGAAGCTACGACAAAAGGAAATGTATGGGTGGGGAGGGCTTGTGGGAGAGTGGTTCAGGTTTCTGA</td><td>140</td><td>6</td><td>WT or Sub</td><td>O</td></tr>
<tr><td>10</td><td>CTTAGCTTATGTCACTGAACACATTTACCAACCCCCCCTCTCTCTACAGCGTGGTTTCTAAGGTTCTTTCCACCGGAAGCTACGACAAAAGGAAATGTATGGGTGGGGAGGGCTTGTGGGAGAGTGGTTCAGGGTTCTGA
||||||||||||||||||||||||||||||||||||||||||||||||||||||||||||||||||||..|||||||||||||||||||||||||||||||.||||||||||||||||||||||||||||||||||||||
CTTAGCTTATGTCACTGAACACATTTACCAACCCCCCCTCTCTCTACAGCGTGGTTTCTAAGGTTCTTGACACCGGAAGCTACGACAAAAGGAAATGTATGTGTGGGGAGGGCTTGTGGGAGAGTGGTTCAGGGTTCTGA</td><td>140</td><td>6</td><td>WT or Sub</td><td>O</td></tr>
</table>
