# Supplementary material for: NFAT1 and NFκB regulates expression of the common γ-chain cytokine receptor in activated T cells
Source: Cell Commun Signal. 2023 Oct 30;21:309. doi: 10.1186/s12964-023-01326-7 (PMC10617197; doi:10.1186/s12964-023-01326-7)
Supplement: Supplementary file 5 — Additional file 4. Full immunoblot images. [file 12964_2023_1326_MOESM4_ESM.pdf]

Fig. 1A

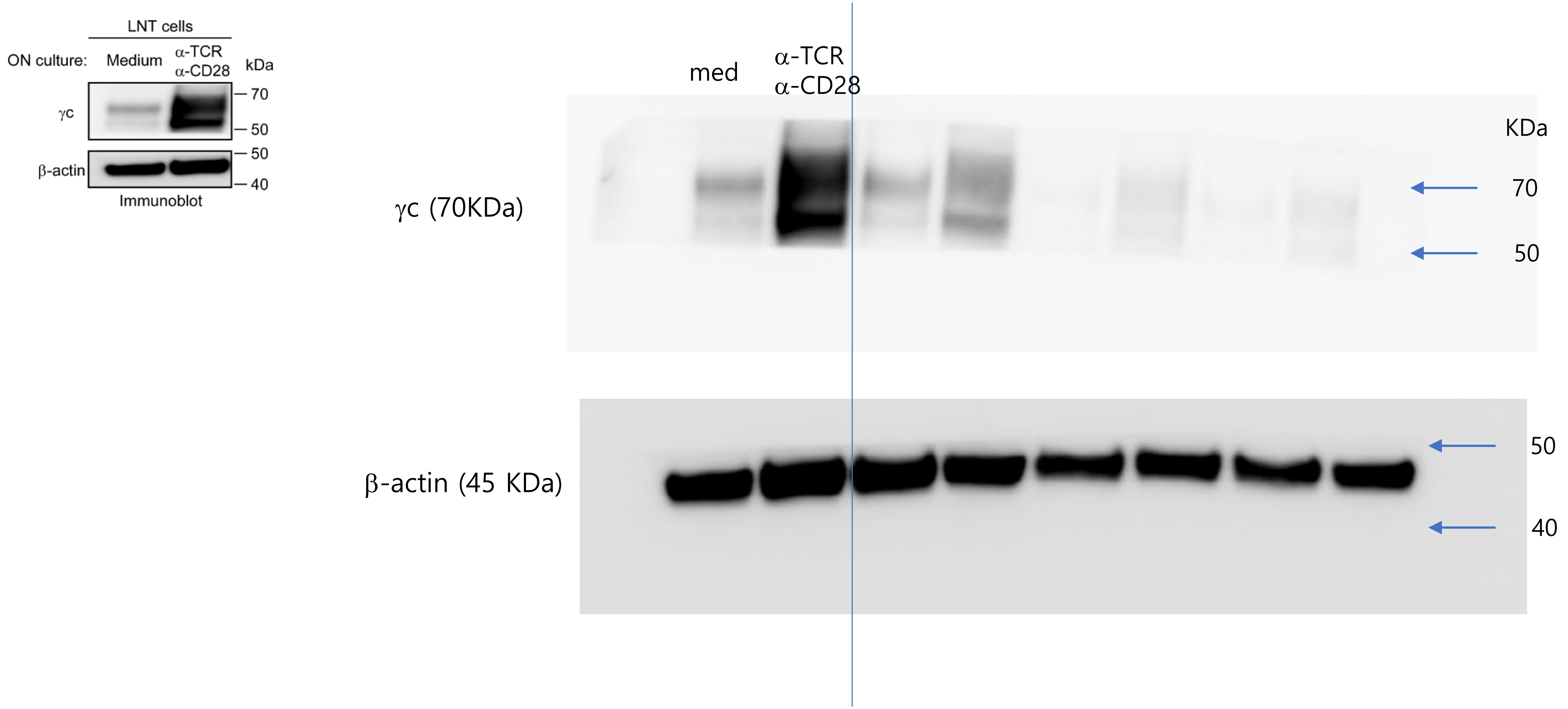

LAS 3000

Fig. 3A

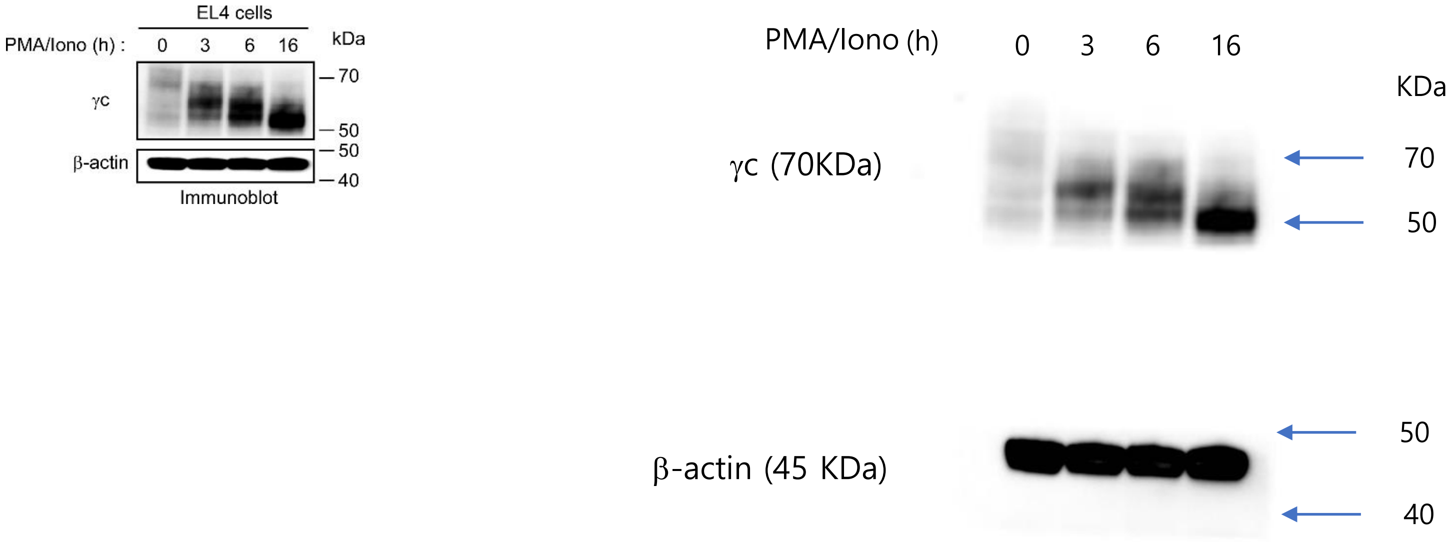
EL4 cells
PMA/Iono (h) : 0 3 6 16
kDa
γc
70
50
β-actin
50
40
Immunoblot
PMA/Iono (h) 0 3 6 16
KDa
γc (70KDa)
70
50
β-actin (45 KDa)
50
40

AI680

Fig. 3E (left)

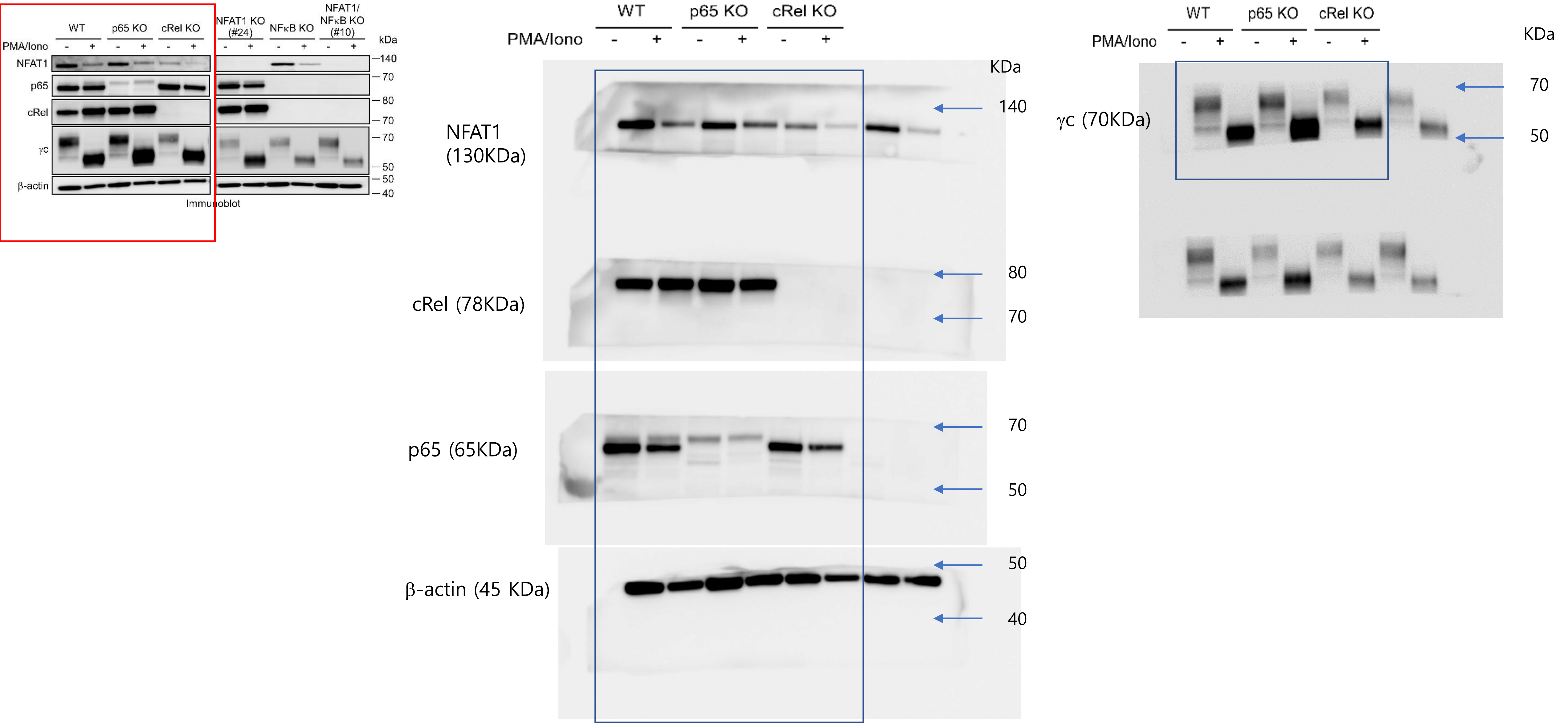

LAS 3000

Fig. 3E (right)

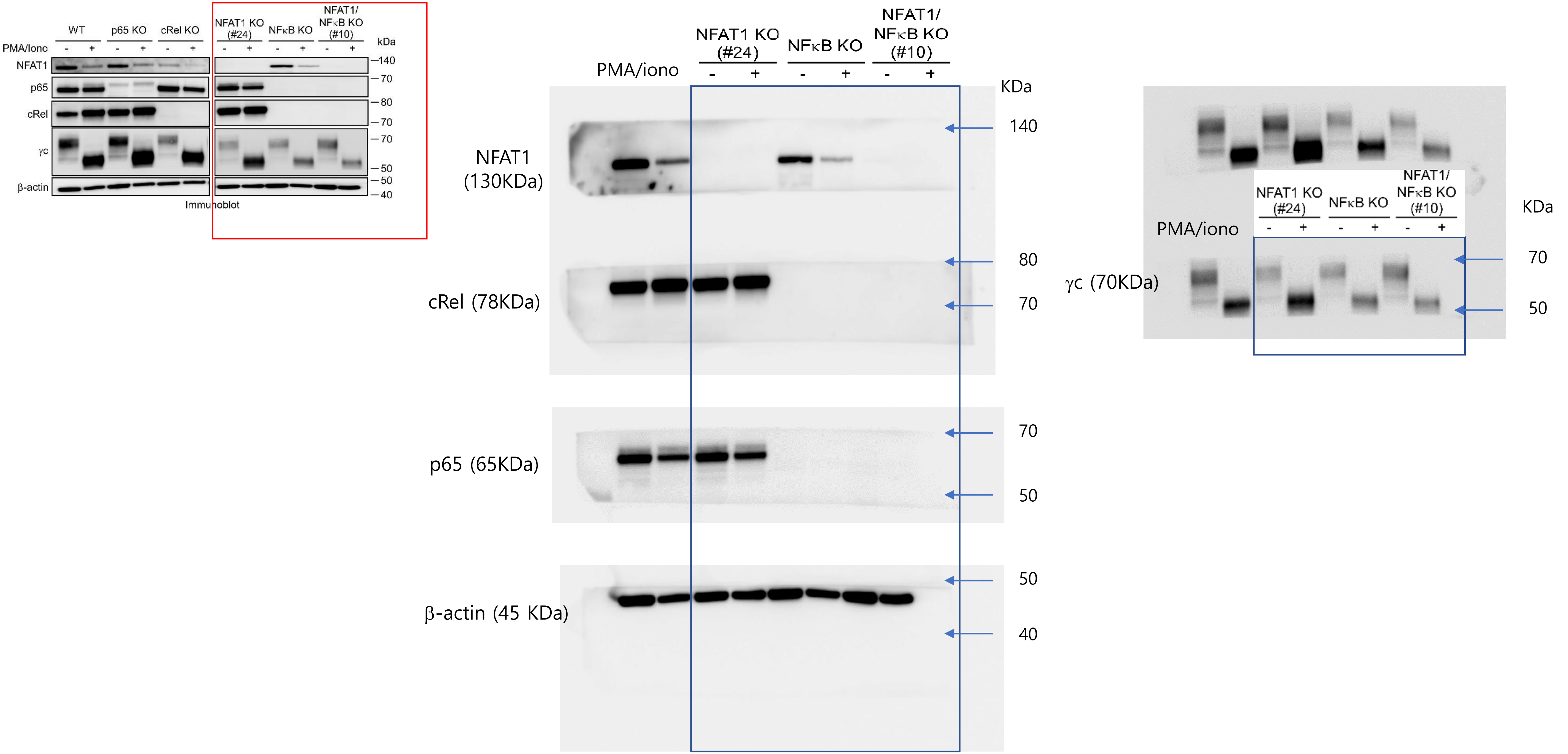

LAS 3000

Fig. 6B

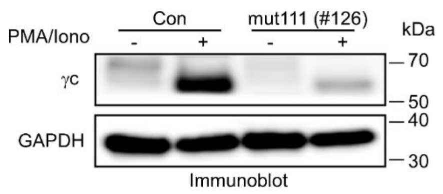
Con
mut111 (#126)
kDa
PMA/Iono
-
+
-
+
γc
70
50
GAPDH
40
30
Immunoblot

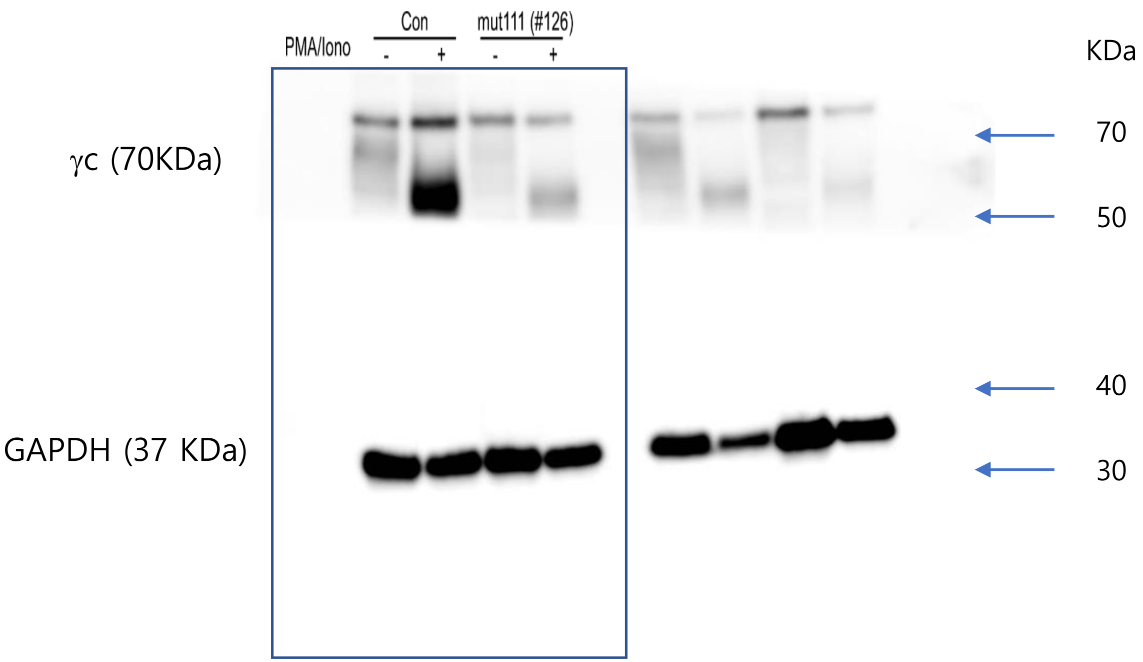
Con
mut111 (#126)
PMA/Iono
-
+
-
+
KDa
γc (70KDa)
70
50
40
GAPDH (37 KDa)
30

AI680

Fig. S1A

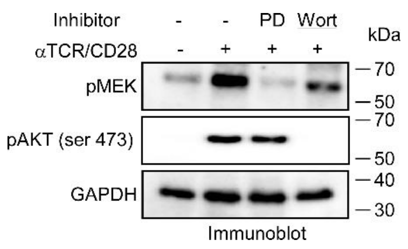

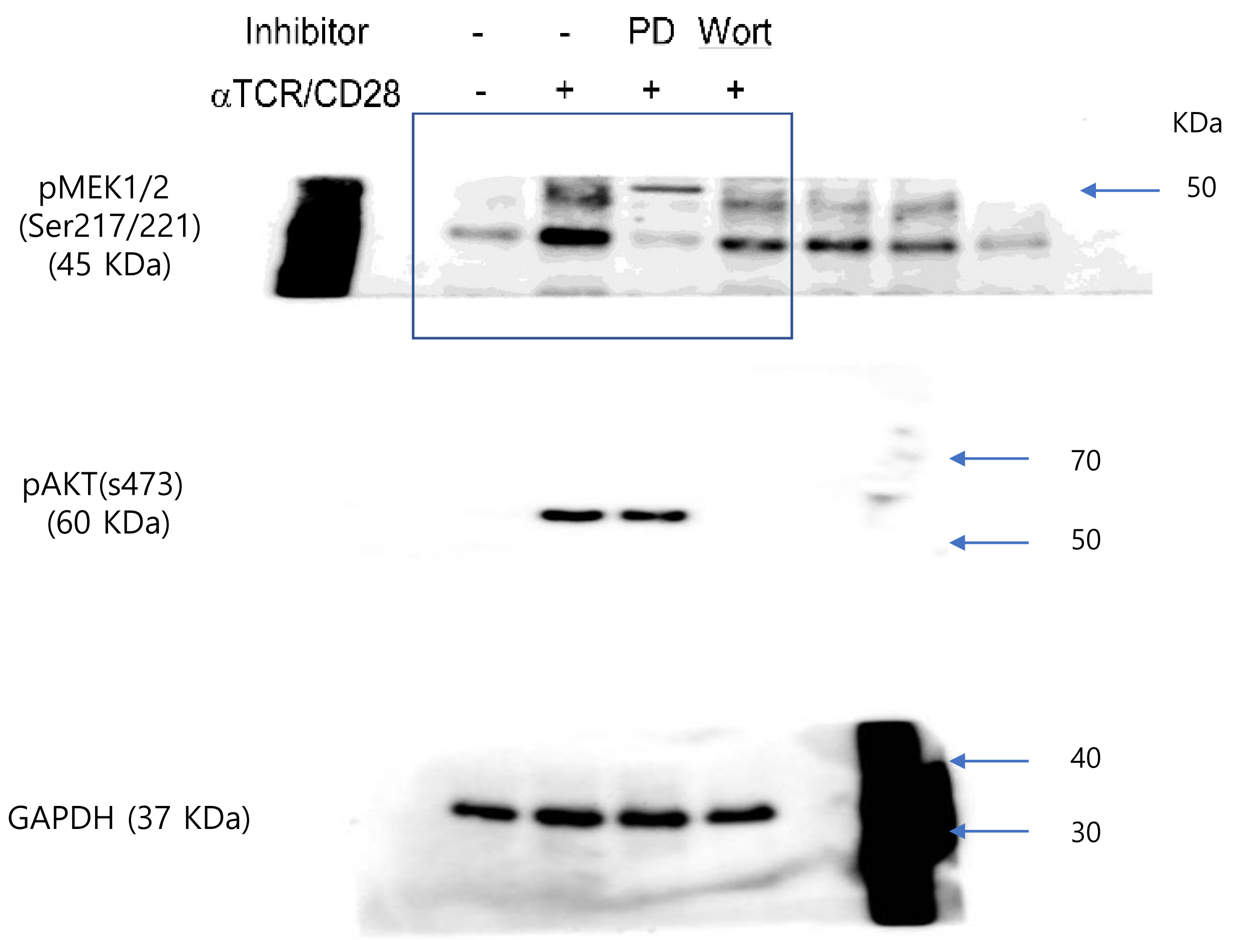

AI680

Fig. S1D

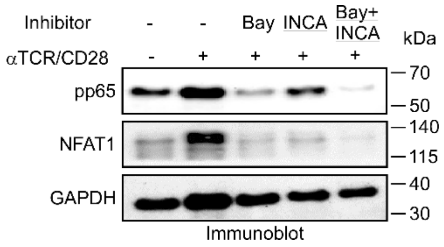

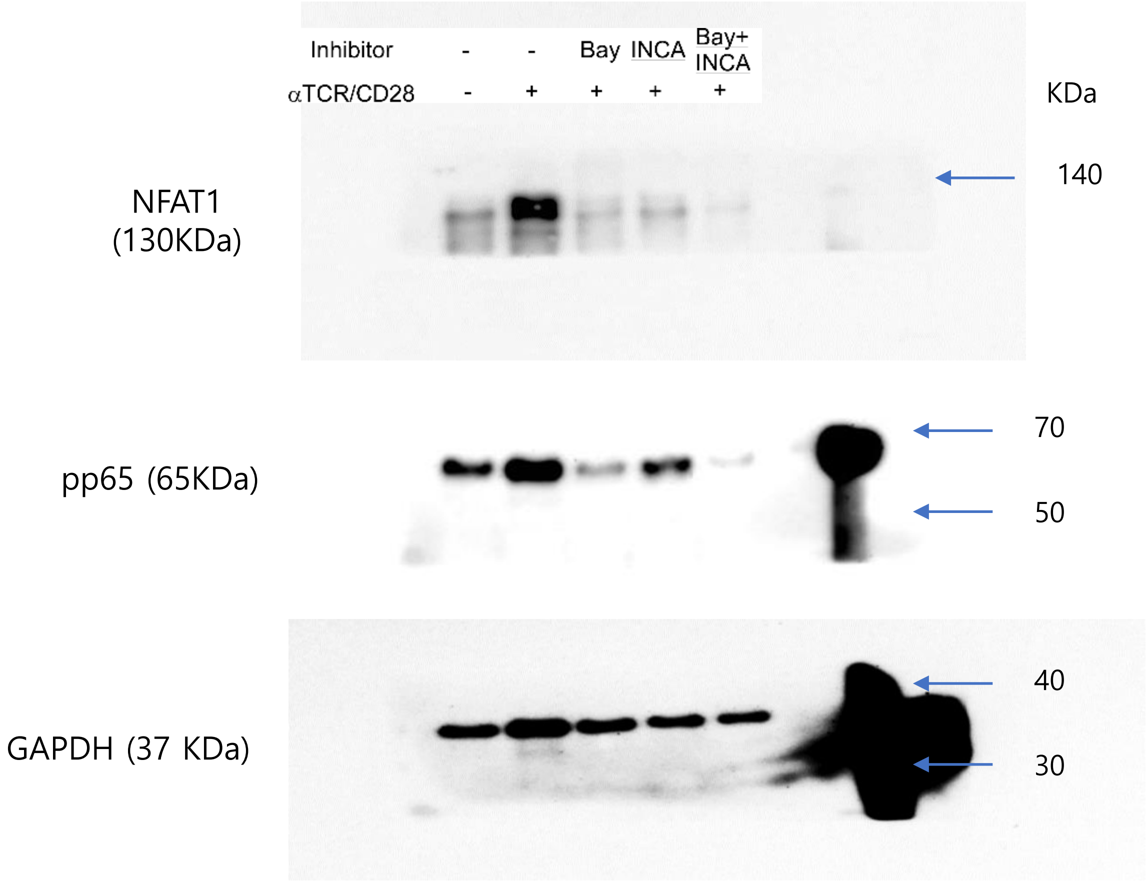

AI680

Fig. S3A

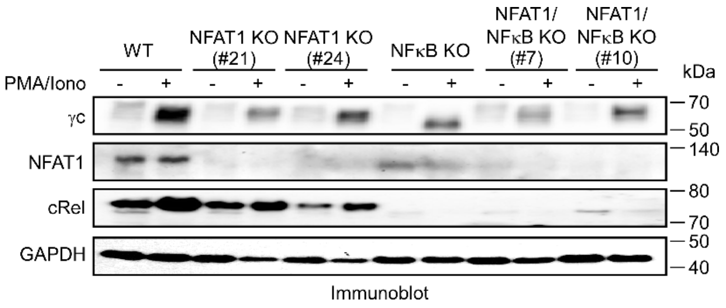

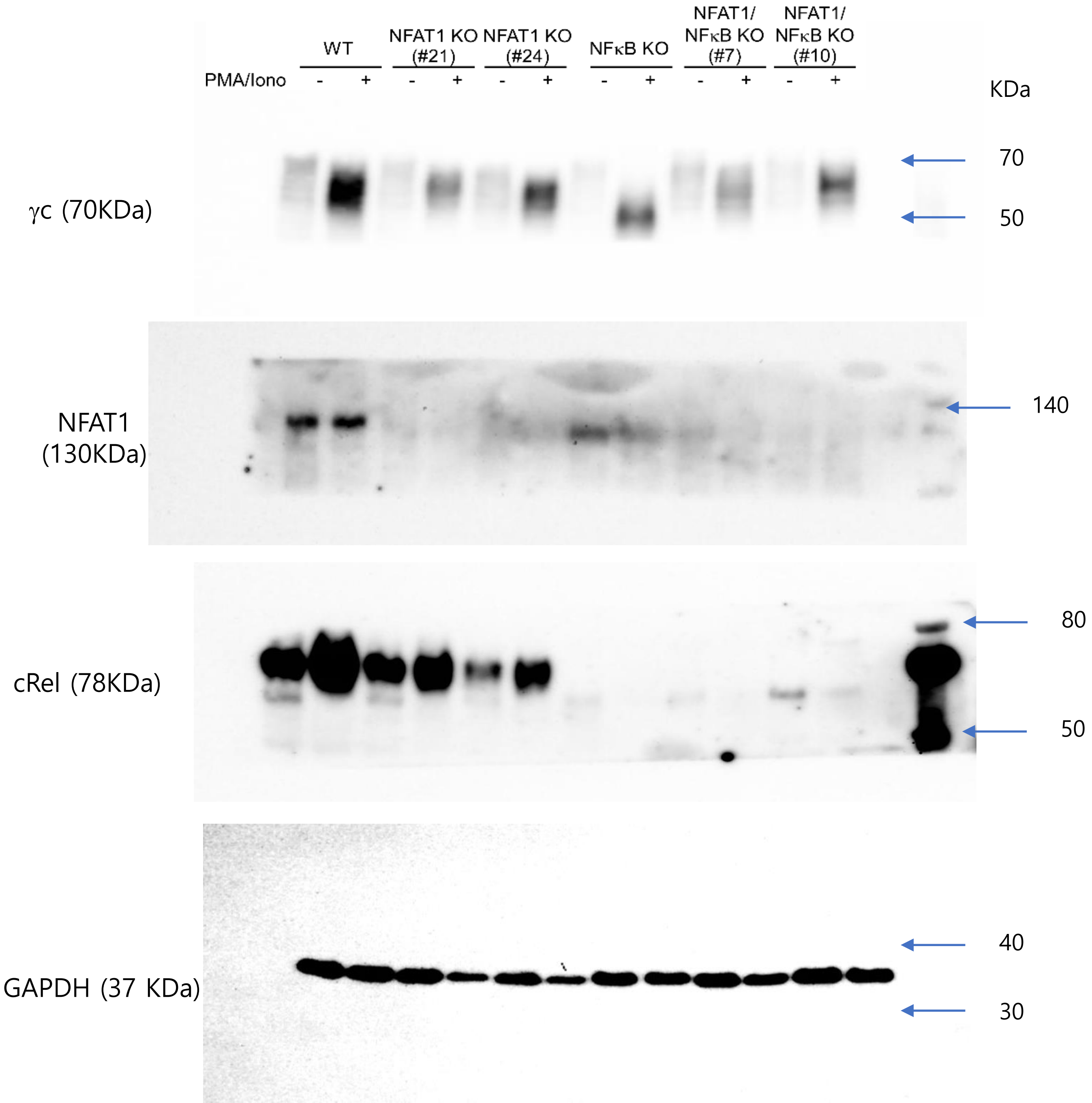

AI680

Fig. S3D

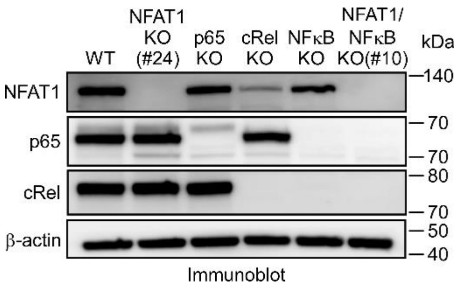

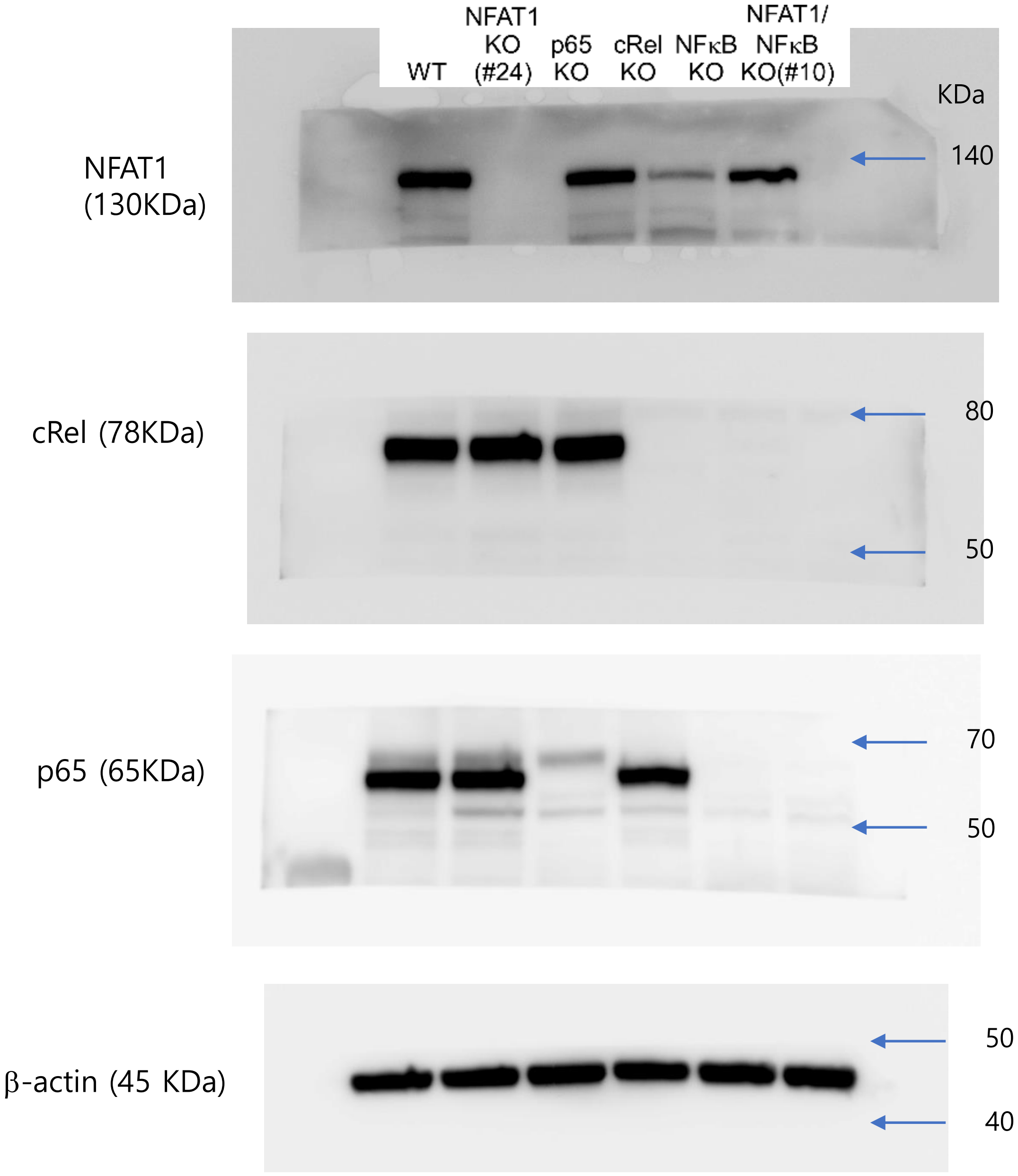

LAS 3000

Fig. S5A

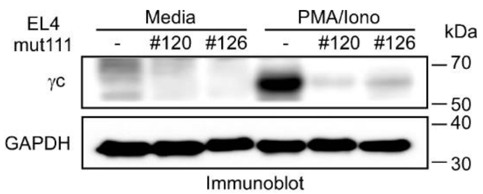

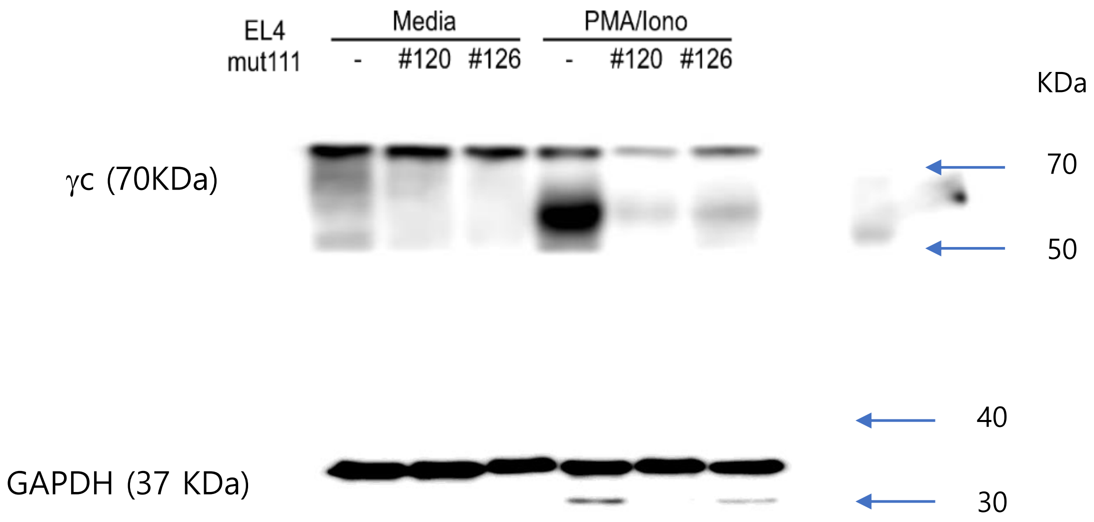

AI680
